# Effectiveness of Non-Pharmacological Interventions for the Prevention of Postoperative Delirium in Critically Ill Adult Patients: A Systematic Review and Meta-Analysis

**DOI:** 10.3390/nursrep16090328

**Published:** 2026-09-08

**Authors:** Matías Fuentealba-Márquez, David Prieto-Merino, Miguel Ángel López-Espinoza

**Affiliations:** 1 Master’s Program in Public Health, Faculty of Health Sciences, Universidad Adventista de Chile, Chillán 3780000, Chile; matiasfuentealba@alu.unach.cl; 2 Coronary Unit, RedSalud Santiago, Santiago 9190015, Chile; 3 Grupo de Investigación en Salud Pública y Epidemiología, Universidad de Alcalá, 28805 Alcalá de Henares, Madrid, Spain; david.prietomerino@uah.es; 4 Centro Regional de Estudios Avanzados en Estilos de Vida Activos y Saludables (CREA-EVAS), Universidad Adventista de Chile, Chillán 3780000, Chile

**Keywords:** postoperative cognitive complications, delirium, non-pharmacological interventions, intensive care units, early ambulation (early mobilization), sleep hygiene, cognitive remediation, sensory aids, music therapy, patient care bundles, systematic review

## Abstract

**Background/Objectives:** Postoperative delirium is a frequent and clinically relevant complication among adult patients admitted to intensive care units (ICUs) following major surgery. It is associated with increased morbidity, prolonged hospitalization, functional decline, and greater healthcare burden. Although non-pharmacological interventions are widely recommended for delirium prevention, evidence regarding their effectiveness in postoperative critically ill adults remains heterogeneous. This systematic review aimed to evaluate the effectiveness of non-pharmacological interventions in preventing postoperative delirium in adult ICU patients. **Methods:** This systematic review was conducted according to PRISMA 2020 guidelines. Searches were performed in PubMed/MEDLINE, Embase, CINAHL, Cochrane CENTRAL, Web of Science, Scopus, LILACS, and Google Scholar. Eligible studies included randomized controlled trials, quasi-experimental studies, and comparative studies evaluating non-pharmacological interventions for postoperative delirium prevention in adult ICU or critical care patients. The primary outcome was postoperative delirium incidence. Secondary outcomes included delirium duration, delirium severity, ICU length of stay, and postoperative sleep quality. Studies reporting sleep-related outcomes without directly assessing delirium were included only in the qualitative synthesis as indirect supportive evidence and were not used to estimate the preventive effect on delirium incidence. **Results:** A total of 26 studies were included in the qualitative synthesis, of which 14 provided sufficient dichotomous data for the primary meta-analysis of delirium incidence. The remaining studies contributed qualitative evidence on non-comparable delirium outcomes, sleep-related outcomes, or the management of established delirium. Non-pharmacological interventions significantly reduced the risk of postoperative delirium compared with control conditions (RR = 0.61; 95% CI: 0.48–0.78; I^2^ = 25.2%). **Conclusions:** Non-pharmacological interventions were associated with a significant reduction in postoperative delirium incidence among critically ill adult patients. The certainty of the evidence for this outcome was moderate, and an analysis restricted to randomized controlled trials produced a consistent result. Further high-quality randomized controlled trials are required to standardize intervention protocols and clarify their effects on secondary clinical outcomes.

## 1. Introduction

Modern surgical practice has progressively improved in terms of safety and clinical outcomes; however, postoperative delirium remains a frequent and clinically significant complication among older adults, particularly following major surgery, where its incidence reaches 19% in elective procedures and 32% in emergency surgery, with variability depending on the type of surgery and the population studied [1]. Delirium is associated with increased mortality, higher rates of complications, prolonged hospital stays, and non-home discharges. Moreover, its timely detection and prevention may reduce the length of hospitalization, decrease pharmacological costs, and enhance functional recovery, findings confirmed in recent reviews that highlight its economic and clinical impact on health systems [2]. For this reason, recognizing delirium as a preventable and treatable event is essential for improving quality of care and patient outcomes in the surgical context, which justifies prioritizing strategies for early detection and prevention as part of preoperative and critical care [1,2].

Between 2020 and 2025, the scientific literature identifies a consistent set of pre-, intra-, and postoperative risk factors that facilitate the identification of high-risk patients: advanced age, frailty or comorbidity, cognitive impairment or a history of delirium, elevated inflammatory markers such as C-reactive protein, high ASA status, low BMI, prolonged surgical and anaesthetic time, and complex postoperative recovery. These factors are useful for risk stratification upon admission to the ICU and for guiding multimodal preventive interventions [3]. Validated tools for systematic detection also exist, such as the CAM-ICU or ICDSC, as well as the RASS for sedation assessment, tools widely used in standard ICU practice and reported in recent clinical guidelines [4].

In the field of non-pharmacological prevention, multicomponent strategies, often implemented by nursing staff, include early mobilisation, sleep hygiene and control of light/noise, cognitive reorientation, optimisation of sensory input, family engagement, and appropriate or light sedation. These strategies are frequently integrated into bundles such as ABCDEF/ICU Liberation, for which evidence demonstrates reductions in delirium and improved clinical outcomes when implemented with high adherence [5,6]. Likewise, nurse-led programmes have shown effectiveness in reducing delirium incidence among hospitalised adults [7], and non-pharmacological nursing interventions have been highlighted as important components of delirium prevention in ICU patients [8].

Sleep-related interventions, including non-pharmacological sleep hygiene bundles, circadian control through light and noise management, eye masks and earplugs, and consolidation of nocturnal care activities, have been associated with reductions in postoperative ICU delirium and with improvements in sleep quality in recent meta-analyses and network comparisons [9].

From a pathophysiological standpoint, an integrative model recognises the interaction between systemic neuroinflammation, blood–brain barrier disruption, neurotransmitter imbalances, neuroendocrine stress, and alterations in circadian rhythm and sleep as key mechanisms precipitating acute brain dysfunction. Non-pharmacological interventions may modulate these mechanisms by reducing systemic inflammatory and stress load, normalising sleep–wake rhythms, stimulating attentional networks, and decreasing the need for deep sedation [10,11].

Other low-risk adjuvant therapies, such as supervised music intervention, are being actively investigated and show potential benefits in reducing the risk of delirium according to recent quantitative syntheses, although certainty remains low and high-quality randomized controlled trials are still required [12].

In this context, postoperative delirium represents a sensitive marker of quality of care and an increasingly relevant public health problem in critical care settings. Its prevention and early detection are shared responsibilities among the interdisciplinary team, with nursing playing a central role in the implementation of structured preventive strategies.

Previous reviews have examined postoperative delirium outcomes, non-pharmacological interventions in ICU patients, nurse-led delirium prevention strategies, sleep-related interventions, and music-based approaches [6,7,8,9,12]. However, the available evidence remains heterogeneous regarding population, surgical context, intervention type, delirium assessment methods, and outcome reporting. Moreover, previous syntheses have not consistently focused on adult postoperative patients admitted to intensive care or critical care settings, nor have they comprehensively compared the range of non-pharmacological interventions used for delirium prevention in this specific clinical context.

Therefore, the aim of this systematic review and meta-analysis was to evaluate the effectiveness of non-pharmacological interventions in preventing postoperative delirium among critically ill adult patients. The primary outcome was the incidence of postoperative delirium. Secondary delirium outcomes included delirium duration, severity, and delirium-free days. Postoperative sleep quality and ICU length of stay were considered supportive clinical outcomes because of their relationship with delirium risk and recovery. Studies that assessed sleep-related outcomes without directly measuring delirium were synthesized separately and were not considered direct evidence of delirium prevention.

Studies involving patients with established delirium were considered only for qualitative assessment of delirium severity or management and were not interpreted as evidence of primary prevention.

## 2. Materials and Methods

### 2.1. Design and Registration

This systematic review and meta-analysis were conducted and reported in accordance with the Preferred Reporting Items for Systematic Reviews and Meta-Analyses (PRISMA 2020) Statement [13]. The review protocol was prospectively registered in the International Prospective Register of Systematic Reviews (PROSPERO; CRD420251247117) on 19 February 2026 (Version 1.0). The PROSPERO record was subsequently updated after completion of the review on 26 August 2026 to document the completed review methods and amendments. Accordingly, classification of analyses as prespecified or post hoc in the present manuscript was based on the prospectively registered Version 1.0 rather than on the subsequent retrospective update.

Version 1.0 contained internally inconsistent entries regarding several eligibility specifications. The overarching PICOS description defined the population as adults aged ≥ 18 years and listed randomized, quasi-experimental, and comparative cohort designs, whereas separate eligibility fields specified an age range of 18–59 years and randomized trials only. Similarly, one eligibility field referred to publications from 2020 to 2025, while the dedicated search section stated that no search-date restrictions would be applied. The final review operationalized these inconsistencies by including adults aged ≥ 18 years without an upper age limit, eligible prospective comparative randomized and non-randomized designs, and a defined search period from 1 January 2014 to 29 May 2026. The lower date limit was selected to focus the review on evidence generated after publication of the 2013 ICU Pain, Agitation, and Delirium guideline framework. These protocol clarifications and deviations are reported in Supplementary Table S7.

The core review question, postoperative delirium incidence as the primary outcome, random-effects meta-analysis, risk ratios for dichotomous outcomes, MDs or SMDs for continuous outcomes, assessment of heterogeneity using I^2^, subgroup analysis according to intervention type, sensitivity analysis excluding studies at high risk of bias, and GRADE certainty assessment were specified prospectively in Version 1.0. Analyses introduced during review completion or peer review are explicitly identified as post hoc or exploratory in Section 2.8 and Supplementary Table S7.

### 2.2. Research Question and PICOS Criteria

The research question that guided this review was: In adults aged ≥ 18 years undergoing major surgery and admitted to an ICU or critical care unit during the postoperative period, do non-pharmacological interventions, compared with standard care, no intervention, historical control, sham intervention, or an alternative intervention, reduce the incidence of postoperative delirium? The PICOS components were defined as follows:

- **Population:** Adults aged ≥ 18 years undergoing major surgery and admitted to an ICU or critical care unit during the postoperative period.
- **Intervention:** Non-pharmacological interventions aimed at preventing postoperative delirium, including multicomponent strategies, early mobilization, sleep hygiene and circadian control, cognitive reorientation, sensory optimization, family engagement, music-based interventions, cerebral monitoring strategies, and structured delirium-prevention or sedation-related protocols.
- **Comparator:** Standard care, usual care, no intervention, historical control, sham intervention, or an alternative intervention.
- **Outcomes:** The primary outcome was the incidence of postoperative delirium, assessed using a validated delirium assessment instrument or clearly defined diagnostic criteria. Secondary delirium outcomes included delirium duration, delirium severity, delirium-free days, and time to delirium onset. Supportive postoperative outcomes included sleep quality, ICU length of stay, hospital length of stay, mechanical ventilation duration, and mortality. Studies reporting only supportive outcomes were retained for qualitative synthesis but were not considered direct evidence of delirium prevention or included in the primary meta-analysis.
- **Study designs:** Randomized controlled trials, pilot randomized controlled trials, cluster-randomized trials, non-randomized controlled clinical trials, prospective non-randomized comparative studies, quasi-experimental studies, and prospective before–after studies with a comparator group.

### 2.3. Inclusion and Exclusion Criteria

Studies were included if they met the following criteria: [1] included adults aged ≥ 18 years admitted to an ICU or critical care setting during the postoperative period; [2] evaluated a preventive non-pharmacological intervention targeting postoperative delirium or a prespecified modifiable delirium-related factor, such as sleep disruption; [3] used a comparator group, including standard care, usual care, historical control, sham intervention, or an alternative intervention; [4] reported at least one direct delirium outcome or a prespecified supportive postoperative outcome; [5] were published in English or Spanish; and [6] were available as full-text publications. Studies reporting supportive outcomes without direct delirium assessment were included only in the qualitative synthesis and were not considered direct evidence of delirium prevention.

Studies were excluded if they focused exclusively on pharmacological interventions, included pediatric or non-critical care populations, addressed delirium outside the postoperative or critical care context, or did not use an eligible comparative design. Narrative reviews, editorials, letters, conference abstracts without full text, interrupted time series analyses, retrospective studies, study protocols without completed clinical outcome data, duplicate publications, and studies with insufficient information to determine eligibility were also excluded. Studies involving patients with established delirium were not considered evidence of primary prevention; when retained, they contributed only to the qualitative synthesis of delirium severity or management outcomes.

As a protocol deviation, a small number of studies enrolling broader critically ill adult ICU populations were retained when the intervention and delirium-related outcome were considered directly relevant to critical-care delirium prevention. The influence of this broader population definition was examined in a post hoc sensitivity analysis restricted to clearly postoperative or surgical populations.

### 2.4. Information Sources and Search Strategy

A comprehensive literature search was conducted in PubMed/MEDLINE through the PubMed interface, Embase via Ovid, CINAHL via EBSCOhost, Cochrane CENTRAL through the Cochrane Library, Web of Science Core Collection, Scopus, LILACS through the Virtual Health Library, and Google Scholar. All information sources were last searched on 29 May 2026. The search strategies combined controlled vocabulary, where applicable, and free-text terms related to delirium, postoperative care, intensive and critical care, and non-pharmacological interventions, including multicomponent bundles, early mobilization, sleep hygiene, circadian control, cognitive reorientation, sensory optimization, family engagement, music therapy, and sedation-related strategies.

For Google Scholar, the predefined search string reported in Supplementary Table S1 was applied with the publication-year restriction from 2014 to 2026. Because Google Scholar ranks results by relevance rather than providing reproducible database-style indexing, results were examined in the order presented by the relevance ranking. The first 320 records returned by the search were retrieved and incorporated into the deduplication and screening workflow. No additional eligibility criteria specific to Google Scholar were applied.

The search was restricted to publications from 1 January 2014 to 29 May 2026. The lower date limit was selected to focus the review on evidence generated within the contemporary framework of delirium prevention and management in adult critical care. In particular, the 2013 Clinical Practice Guidelines for the Management of Pain, Agitation, and Delirium in Adult Patients in the ICU [14] represented an important transition toward an integrated, evidence-based approach to delirium assessment and prevention, including closer integration with sedation practices, early mobility, and sleep-related interventions. Accordingly, 2014 was selected as the beginning of the search period to capture evidence generated after the introduction of this contemporary critical-care framework. We acknowledge, however, that this time restriction may have resulted in the exclusion of potentially relevant earlier studies. Studies published in English or Spanish and available as full-text publications were considered eligible. All retrieved records were exported to Rayyan QCRI for reference management, duplicate removal, and study screening [15].

The complete search strategies, database platforms, search limits, search dates, and the Google Scholar search procedure are provided in Supplementary Table S1.

### 2.5. Selection Process

The study selection process was conducted independently by two reviewers (M.F.-M. and M.Á.L.-E.). First, titles and abstracts were screened according to the predefined eligibility criteria. Potentially relevant studies were subsequently retrieved and assessed in full text. Studies were classified as included, excluded, or uncertain according to the eligibility criteria. Any disagreements between the two reviewers were resolved through discussion and consensus. When consensus could not be reached, a third reviewer (D.P.-M.) was consulted to make the final decision. The study selection process was summarized using the PRISMA 2020 flow diagram [13].

### 2.6. Data Extraction

Data were extracted by one reviewer (M.F.-M.) and checked against the primary source reports by a second reviewer (M.Á.L.-E.). For each included study, information was collected on the first author, year of publication, country, study design, clinical population and setting, total and group-specific sample size, participant age and sex, intervention components, intervention frequency and duration, comparator content, delirium assessment instrument and assessment schedule, follow-up period, primary and secondary outcomes, and the main numerical or qualitative findings. Information was extracted from the primary study report and associated Supplementary Materials, when available. Characteristics that were not reported in the primary publication or its Supplementary Materials were recorded as not reported (NR) and were not imputed. Any discrepancies identified during checking were discussed and resolved by consensus.

### 2.7. Risk of Bias Assessment Methods

Risk of bias was assessed according to study design. Randomized controlled trials were evaluated using the Cochrane Risk of Bias 2 tool (RoB 2) [16,17], which assesses bias arising from the randomization process, deviations from intended interventions, missing outcome data, measurement of the outcome, and selection of the reported result. Non-randomized studies were assessed using the Risk of Bias in Non-randomized Studies of Interventions tool (ROBINS-I) [18], which evaluates bias due to confounding, selection of participants, classification of interventions, deviations from intended interventions, missing data, measurement of outcomes, and selection of the reported result.

RoB 2 was applied to 21 randomized studies, whereas ROBINS-I was applied to five non-randomized studies: Guo et al. (2016) [19], Patel et al. (2014) [20], Welsch et al. (2023) [21], Zhang et al. (2017) [22], and Chen et al. (2021) [23]. Domain-level and overall judgments were generated according to the criteria of the corresponding assessment tool, and visual summaries were produced using the ROBVIS web application [24].

Risk-of-bias assessments were performed independently by two reviewers (M.F.-M. and M.Á.L.-E.). Disagreements were resolved through discussion and consensus, with consultation of a third reviewer (D.P.-M.) when required.

The prospectively registered protocol referred to Cochrane RoB 1 and RoB 2. In the final review, RoB 2 was used for randomized trials and ROBINS-I for non-randomized intervention studies because ROBINS-I provides a design-appropriate assessment of confounding and other sources of bias specific to non-randomized studies. This represents a methodological update from the registered risk-of-bias plan.

### 2.8. Statistical Analysis

Meta-analyses were conducted using R version 4.6.1 (R Foundation for Statistical Computing, Vienna, Austria) [25] and the meta package version 8.5-0 [26]. Random-effects models were selected a priori because clinical and methodological heterogeneity was expected across populations, interventions, and outcome-assessment methods. For dichotomous outcomes, risk ratios (RRs) were pooled on the logarithmic scale using inverse-variance weighting. Between-study variance was estimated using the restricted maximum likelihood method (REML). For studies containing a zero cell, a continuity correction of 0.5 was added to all four cells of the corresponding 2 × 2 table. Conventional Wald-type 95% confidence intervals were used for the prespecified primary analysis. In response to peer-review concerns regarding uncertainty in random-effects inference, an additional post hoc sensitivity analysis was conducted using the Hartung–Knapp adjustment while retaining the REML estimate of between-study variance [27].

For continuous outcomes, mean differences (MDs) were used when studies reported outcomes on a common scale, whereas standardized mean differences (SMDs) were used when different measurement scales were employed. When continuous outcomes were reported as medians and interquartile ranges, means and standard deviations were estimated using the methods described by Wan et al. [28]. Statistical heterogeneity was quantified using the I^2^ statistic and the between-study variance estimate (τ^2^).

Although the included interventions differed in their specific components, they were considered clinically suitable for the primary synthesis because they represented non-pharmacological preventive strategies targeting modifiable precipitating factors for postoperative delirium and evaluated the same primary outcome, incident postoperative delirium. The random-effects model was therefore interpreted as estimating the average effect across this broader class of preventive interventions rather than assuming an identical intervention effect across strategies. Intervention category was considered a potential effect modifier and was explored through subgroup analysis.

Eligibility for the primary meta-analysis was determined by the availability of comparable dichotomous data for incident postoperative delirium and was independent of the magnitude, direction, or statistical significance of individual study findings. Studies that did not provide compatible incidence data were retained in the qualitative synthesis rather than excluded from the review.

For the revised quantitative synthesis, one independent effect estimate was retained per study population. When a study reported more than one delirium result, an explicit outcome-selection hierarchy was applied. First, we preferentially extracted the study-defined cumulative incidence of delirium over the full prespecified postoperative observation period. When different validated instruments were used according to the clinical setting or level of consciousness (e.g., CAM and CAM-ICU), the study-defined combined diagnostic outcome was retained rather than treating each instrument as a separate outcome. When an initial screening instrument was followed by confirmatory diagnostic assessment, the final confirmed delirium diagnosis was used. When only separate timepoint-specific binary results were available and cumulative incidence could not be derived, the latest prespecified postoperative assessment was selected, provided that it represented postoperative delirium in participants eligible for primary prevention. Studies for which new incident delirium could not be distinguished from delirium or confusion already present at baseline were not included in the incidence meta-analysis. Similarly, studies reporting delirium severity, delirium/coma-free days, or other continuous or ordinal delirium outcomes without an extractable dichotomous incidence estimate were retained in the qualitative or secondary-outcome synthesis but were not converted to incidence data. For multi-arm trials containing more than one eligible non-pharmacological intervention arm and a shared control group, eligible intervention arms were combined to provide a single study-level comparison and avoid double-counting the control group. Outcome selection was performed independently of the magnitude, direction, or statistical significance of the reported effects.

Prespecified analyses included the primary random-effects meta-analysis of delirium incidence, use of risk ratios for dichotomous outcomes and MDs or SMDs for continuous outcomes, assessment of heterogeneity using I^2^, subgroup analysis according to intervention type, sensitivity analysis excluding studies at high risk of bias, and assessment of certainty using GRADE. PROSPERO also proposed subgroup analyses according to type of surgery, participant age, frailty, and intervention adherence. These additional subgroup analyses were not performed because the relevant characteristics were inconsistently reported and the number of studies within potential strata was insufficient for reliable quantitative comparisons.

Post hoc analyses included the formal outcome-selection hierarchy for studies reporting multiple delirium instruments or timepoints, leave-one-out influence analysis, sensitivity analysis restricted to randomized controlled trials, stratification according to RoB 2 overall judgment, Hartung–Knapp adjustment, Egger’s regression test using the *p* < 0.10 interpretive threshold, Duval–Tweedie trim-and-fill analysis, and the sensitivity analysis restricted to clearly postoperative or surgical populations. These analyses were used to examine robustness and potential sources of uncertainty and did not replace the primary random-effects analysis.

Subgroup analyses were conducted according to intervention category for the primary outcome. Potential small-study effects and reporting bias were assessed through visual inspection of the funnel plot and Egger’s regression test because at least ten studies were available. In response to peer-review concerns regarding the limited power of funnel-plot asymmetry tests, a two-sided significance threshold of *p* < 0.10 was applied to Egger’s test. Funnel-plot asymmetry was interpreted as evidence of possible small-study effects rather than definitive evidence of publication bias. An exploratory Duval–Tweedie trim-and-fill analysis was additionally performed to examine the sensitivity of the pooled estimate to a hypothetical pattern of missing studies [29]. Sensitivity analyses included a leave-one-out analysis and an additional analysis restricted to randomized controlled trials, excluding studies with non-randomized designs. Both sensitivity analyses used the same random-effects model and statistical specifications as the primary analysis. The prespecified sensitivity analysis according to methodological quality was operationalized by excluding randomized trials judged to have high overall risk of bias using RoB 2 and non-randomized studies judged to have serious overall risk of bias using ROBINS-I. In addition, exploratory subgroup analyses were performed among randomized controlled trials according to their overall RoB 2 classification (low risk, some concerns, or high risk). The certainty of the evidence for postoperative delirium incidence was assessed using the GRADE approach.

## 3. Results

### 3.1. Study Selection

A total of 3615 records were identified through database searching. The records were retrieved from PubMed/MEDLINE (*n* = 620), Embase (*n* = 580), CINAHL (*n* = 420), Scopus (*n* = 690), Web of Science (*n* = 510), Cochrane CENTRAL (*n* = 295), LILACS (*n* = 180), and Google Scholar (*n* = 320). After removing 1296 duplicate records, 2319 records were screened by title and abstract. Of these, 2211 records were excluded because they were not related to postoperative delirium prevention, involved non-critical care or non-postoperative populations, evaluated pharmacological interventions, or did not meet the predefined eligibility criteria.

A total of 108 reports were sought for retrieval, and all were retrieved for full-text assessment. After full-text review, 82 reports were excluded for the following reasons: wrong study design (*n* = 19), wrong outcome (*n* = 24), wrong intervention (*n* = 15), wrong population (*n* = 11), wrong setting (*n* = 4), duplicate article (*n* = 2), wrong delirium assessment method (*n* = 1), and protocol reports not meeting eligibility criteria (*n* = 6). The reports excluded after full-text assessment and the corresponding reasons for exclusion are presented in Supplementary Table S4. Finally, 26 studies met the inclusion criteria and were included in the qualitative synthesis.

Among the 26 included studies, 14 provided sufficient dichotomous data on postoperative delirium incidence and were included in the primary meta-analysis. The remaining 12 studies were retained in the qualitative synthesis because they reported non-comparable delirium outcomes, supportive postoperative outcomes such as sleep quality, or outcomes related to the severity or management of established delirium. These studies were not used to estimate the pooled preventive effect on delirium incidence.

**Figure 1:**
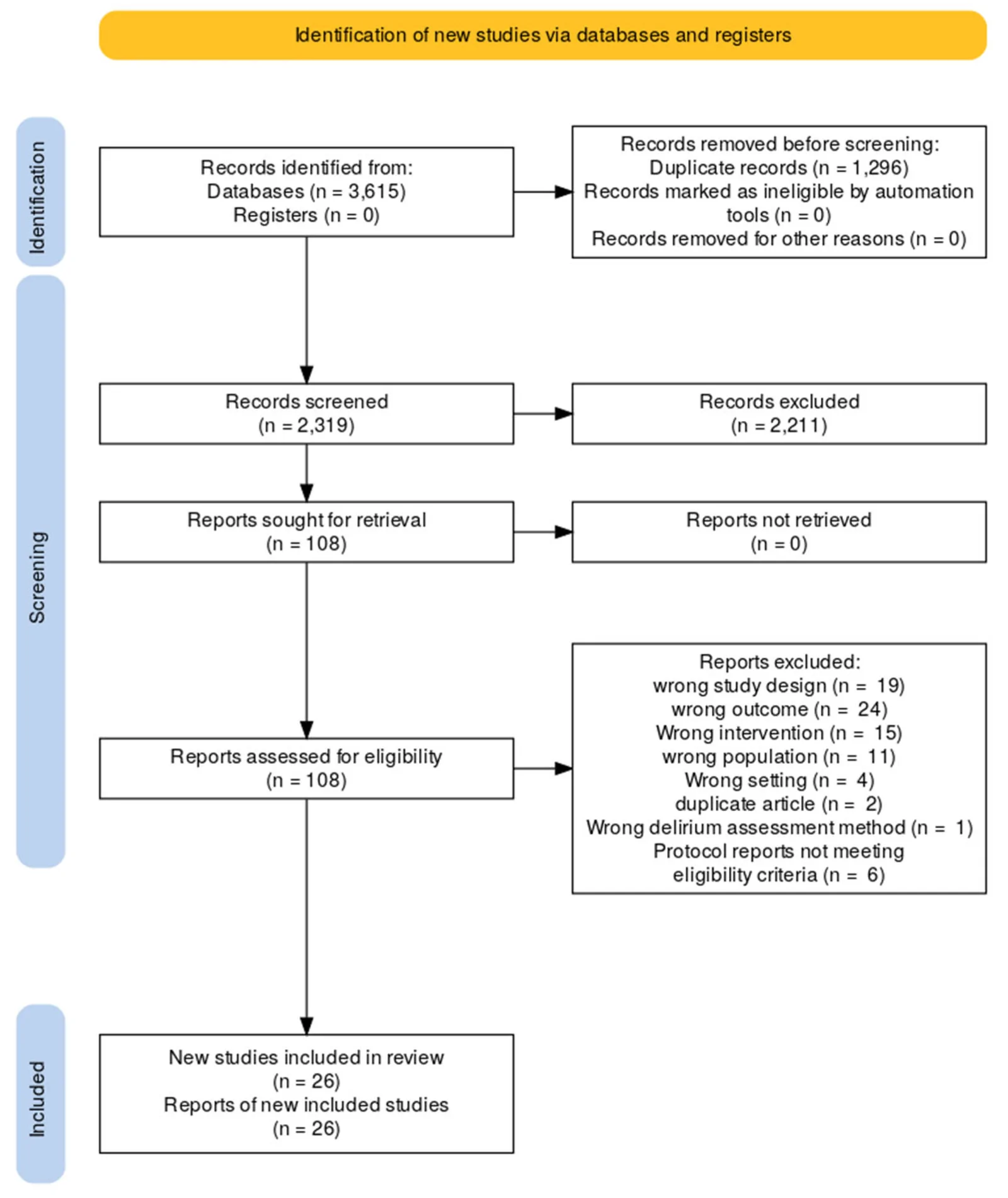
PRISMA 2020 flow diagram of the study selection process generated using Rayyan. The labels “new studies included in review” and “reports of new included studies” are automatically generated by the software; in this review, both refer to the 26 studies and corresponding reports included in the qualitative synthesis. A total of 3615 records were identified, 108 reports were assessed for eligibility, and 82 reports were excluded after full-text assessment. Fourteen of the 26 included studies provided sufficient dichotomous data for the primary meta-analysis of postoperative delirium incidence.

The complete study selection process is presented in Figure 1.

### 3.2. Characteristics of Included Studies

The 26 studies included in the systematic review were published between 2014 and 2026. Most studies were randomized controlled trials (RCTs) (*n* = 21), including pilot and cluster-randomized designs. Five studies used non-randomized designs, including prospective non-randomized, before–after, comparative, and quasi-experimental designs.

Geographically, the studies were conducted across 12 countries. China contributed the largest number of studies (*n* = 9), followed by Türkiye (*n* = 3), Iran (*n* = 2), South Korea (*n* = 2), the United Kingdom (*n* = 2), and the United States (*n* = 2). One study was conducted in each of the Netherlands, Spain, Thailand, Canada, Colombia, and Brazil.

Sample sizes varied substantially across the included studies, ranging from small pilot trials of approximately 30 participants to studies including more than 500 participants. Most populations consisted of middle-aged or older adults, although age thresholds and demographic reporting varied across studies. Intervention exposure was also heterogeneous, ranging from single perioperative or overnight sessions to interventions administered twice daily, for up to seven consecutive days, or throughout the ICU stay. Comparator conditions included routine or usual care, standard monitoring, historical controls, sham stimulation, an attention-control audiobook, and absence of overnight family presence. Delirium assessment schedules ranged from early single postoperative reassessments to twice-daily monitoring for up to seven days or surveillance throughout ICU admission. Detailed total and group-specific sample sizes, available age and sex distributions, intervention components, frequency and duration, comparator content, assessment timing, and follow-up periods are reported in Table 1.

The included populations primarily consisted of adult and older postoperative patients admitted to intensive care units or critical care settings following cardiac, abdominal, spinal, colorectal, laparoscopic, hip, or mixed surgical procedures. Some studies also included broader adult ICU populations or mechanically ventilated critically ill patients when the intervention and delirium-related outcomes were relevant to postoperative or critical care delirium prevention.

Regarding outcome assessment, most included studies directly evaluated delirium incidence, severity, duration, or related delirium measures using validated instruments. However, two studies—Hu et al. (2015) [30] and Chen et al. (2021) [23]—primarily evaluated postoperative sleep quality or sleep-related environmental outcomes without directly measuring delirium and were therefore retained as indirect supportive evidence. Kim et al. (2020) [31] primarily evaluated postoperative sleep quality and neuroendocrine outcomes but also assessed postoperative delirium during the ICU stay using a validated delirium assessment instrument. Erbay Dallı et al. (2023) [32] enrolled patients with established delirium and evaluated changes in delirium severity and related clinical outcomes; therefore, it was considered evidence related to delirium management rather than primary prevention.

A broad range of non-pharmacological interventions was evaluated. Using mutually exclusive intervention categories, music-based or combined music and sensory interventions were the most frequently studied strategies (*n* = 7), followed by multicomponent delirium-prevention bundles or tailored preventive protocols (*n* = 6), family-engagement interventions (*n* = 3), cognitive or educational interventions (*n* = 2), sleep-promotion strategies (*n* = 2), and nurse-led interventions targeting delirium risk factors (*n* = 2). Early mobilization, cerebral oximetry monitoring, mirror intervention, and transcutaneous acupoint electrical stimulation were each evaluated in one study (*n* = 1).

However, the outcomes evaluated differed across the intervention categories. Hu et al. (2015) [30] and Chen et al. (2021) [23] primarily assessed postoperative sleep quality or sleep-related environmental outcomes without directly measuring incident delirium and were therefore considered indirect supportive evidence. Kim et al. (2020) [31] primarily focused on postoperative sleep quality but additionally assessed postoperative delirium during the ICU stay; its eligibility for quantitative synthesis of delirium incidence was therefore evaluated separately according to the outcome-selection hierarchy described in Section 2.8. In addition, Erbay Dallı et al. (2023) [32] evaluated a music intervention in mechanically ventilated ICU patients with established delirium and contributed evidence on delirium severity and management rather than primary prevention.

Delirium was most commonly assessed using the Confusion Assessment Method for the Intensive Care Unit (CAM-ICU). Other tools included the Confusion Assessment Method (CAM), Nursing Delirium Screening Scale (Nu-DESC), Delirium Observation Screening Scale (DOSS), CAM-ICU-7, Delirium Detection Score (DDS), 4AT, and the Intensive Care Delirium Screening Checklist (ICDSC). One study additionally confirmed delirium using DSM-5 diagnostic criteria. For the studies that did not directly assess delirium, postoperative sleep quality was evaluated using the Richards–Campbell Sleep Questionnaire, together with physiological or environmental measurements such as melatonin, cortisol, noise, and light exposure.

**Table 1:** Detailed characteristics of studies included in the systematic review (*n* = 26).

Study/Design/Country	Population and Sample Characteristics	Intervention: Components, Dose and Duration	Comparator	Delirium/Related Assessment and Follow-Up
Topal et al. (2025) [33] RCT; Türkiye	Abdominal surgery patients in general surgery ICU. N = 45: intervention 21, control 24. Age: 63.23 ± 15.4 vs. 61.29 ± 13.5 y. Female: 9 vs. 8.	Eye mask beginning at 22:00 plus 1 h of calming classical music nightly for 3 consecutive nights.	Standard clinical ICU routine without study intervention.	Nu-DESC at 22:00 and RCSQ at 10:00 during the 3-day study period; RASS also recorded.
Jiang et al. (2024) [34] Multicenter RCT; China	Elective CABG. 218 randomized; 208 analyzed: 102 cognitive training, 106 routine care. Age: 65 (IQR 58–70) vs. 66 (IQR 58–70) y. Female: 25/102 vs. 39/106.	Preoperative online cognitive exercises involving memory, imagination, reasoning, reaction time, attention and processing speed. Exposure 1–9 h; median 6 (IQR 5–7) h; ≥3 h defined as adherence. Patients enrolled ≥ 10 d before surgery.	Routine perioperative care.	CAM/CAM-ICU. Delirium assessed POD1–7 or until hospital discharge; mortality followed to 30 d.
Kappen et al. (2023) [35] RCT; Netherlands	Adults undergoing craniotomy. N = 189: music 95, control 94; 184 constituted the modified ITT population. Median age approximately 60 y; 44% female overall.	Perioperative patient-preferred music administered before, during and after surgery and continued during the early postoperative period.	Standard clinical care without study music.	Daily DOSS; DOSS threshold triggered DSM-5 psychiatric confirmation. Primary delirium observation window: first 5 postoperative days.
Cao et al. (2023) [36] RCT; China	Patients after PCI for acute myocardial infarction; N = 148 (intervention 75, control 73). Age: NR; sex: NR.	Routine nursing plus family-visit nursing incorporating emotional support, patient education and enhanced communication with family members.	Conventional nursing care.	RASS and CAM-ICU; assessments at 24 and 48 h after PCI; delirium incidence and duration reported.
Méndez-Martínez et al. (2024) [37] Double-blind RCT; Spain	Cardiac surgery patients. N = 104: 52/52. Overall, 66.35% male. Age: NR.	Visual projection of photographs/material involving relatives and loved ones during the immediate postoperative ICU period.	Usual postoperative ICU treatment.	CAM-ICU; baseline evaluation 30 min after extubation and post-intervention follow-up assessment.
Xu et al. (2026) [38] Pilot RCT; China	Adult post-cardiac-surgery patients. N = 60: 30/30. Age: NR; sex: NR.	Unsupported sitting in a wheelchair for >30 min within 24 h of ICU admission, repeated daily through day 7.	Mobilization initiated after transfer from ICU to the cardiac surgery ward, then repeated daily through day 7.	CAM-ICU during anesthesia recovery, 8 h after awakening, POD2 and every morning thereafter. Primary follow-up: first 7 d.
Moon et al. (2015) [39] Single-blind RCT; South Korea	Adult ICU patients. N = 123: intervention 60, control 63. Age: NR; sex: NR.	Tailored multicomponent delirium-prevention protocol applied daily during the first 7 ICU days.	Routine ICU care.	Delirium and ICU outcomes assessed during hospitalization; mortality evaluated at 7 and 30 d.
Gómez Tovar et al. (2024) [40] Double-masked RCT; Colombia	Critically ill adults. N = 213: intervention 71, control 142. Mean age 60.3 ± 15.2 y; 59.6% male.	DyDel multicomponent nursing intervention addressing physiological, psychological, spiritual and social needs, administered every nursing shift from day 0 until ICU discharge.	Daily/usual ICU care.	Participants followed each shift for delirium incidence and duration until ICU discharge.
Khan et al. (2020) [41] Pilot RCT; USA	Mechanically ventilated medical-surgical ICU patients. N = 52: personalized music 17, slow-tempo music 17, audiobook 18. Mean age 57.4 ± 14.2 y; 52% female.	Personalized or relaxing slow-tempo music through noise-cancelling headphones, 1 h twice daily for up to 7 d.	Audiobook attention-control sessions of equivalent duration.	CAM-ICU and CAM-ICU-7 twice daily; delirium/coma-free days evaluated through day 7.
Guo et al. (2016) [19] Comparative clinical study; China	N = 122 postoperative ICU patients. Age: NR; sex: NR.	Preoperative nurse-led/multidisciplinary standardized protocol addressing staff education, cognitive care, environmental safety, social support and sleep optimization.	Usual care.	Delirium Detection Score at multiple postoperative timepoints; delirium severity specifically evaluated within the first 24 h.
Abbasinia et al. (2021) [42] Single-blind RCT; Iran	CABG ICU patients. N = 60: 30/30. Age: intervention 56.46 ± 9.89 vs. control 58.93 ± 10.57 y. Male: 16/30 vs. 17/30.	Routine care plus educational video and tailored intervention based on the Hospital Elder Life Program (HELP).	Routine postoperative care.	CAM-ICU and RASS; outcomes evaluated on POD2 and POD3.
Giraud et al. (2016) [43] Pilot time-cluster RCT; UK	Cardiac surgical ICU patients ≥ 70 y. N = 223: mirrors 115, usual care 108. Age: 77.4 ± 4.8 vs. 77.0 ± 4.9 y. Approximately 24% female overall.	Structured use of personal/posture mirrors at protocol-defined clinical times during ICU admission.	Standard cardiac-surgical ICU care without prescribed mirror intervention.	Delirium assessed throughout ICU stay. ICU outcomes assessed at discharge; factual/delusional memory and HRQoL followed at 12 wk.
Welsch et al. (2023) [21] Prospective non-randomized pilot; USA	Postoperative spine ICU. N = 30: family presence 16, unaccompanied 14. Age: NR; sex: NR.	A family member remained with the patient during the first postoperative night.	No family member remained overnight.	RASS, CAM-ICU, 4AT and GCS orientation collected after ICU admission and again morning of POD1.
Patel et al. (2014) [20] Before-after study; UK	Mixed medical/surgical ICU. N = 338: pre-intervention 167, post-intervention 171. Age: 60.0 ± 13.7 vs. 60.6 ± 16.3 y; male 51% vs. 53%.	Multicomponent nighttime bundle reducing environmental noise/light and unnecessary overnight care interruptions; compliance >90%.	Historical pre-implementation ICU care.	Delirium monitored during ICU admission; incidence and delirium duration assessed.
Lei et al. (2017) [44] Double-blind RCT; Canada	Cardiac surgery with cardiopulmonary bypass; age ≥ 60 y. N = 249: intervention 123, control 126. Sex: NR.	Cerebral oximetry with a corrective algorithm activated when regional cerebral oxygen saturation fell <75% of baseline for ≥1 min.	Cerebral-oximetry display electronically blinded; standard intraoperative monitoring.	CAM-ICU while in ICU and CAM after ICU discharge, assessed every 12 h for 7 postoperative days.
Kim et al. (2020) [31] RCT; South Korea	Older postoperative ICU patients. N = 133: interactive music therapy 44, passive listening 44, and control 45. Age: 74.6 ± 5.2, 72.3 ± 4.7, and 74.1 ± 6.7 years, respectively. Sex (M: F): 31:13, 32:12, and 35:10, respectively.	ICU day 1: interactive music therapy up to 20 min, or passive preselected relaxing music for 30 min.	Routine medical care.	RCSQ and QoR40 assessed on POD1 and POD2; salivary melatonin and cortisol assessed perioperatively. Patients were screened for postoperative delirium three times daily during ICU stay using CAM-ICU; postoperative delirium was additionally reported as a binary clinical outcome (ICDSC score ≥ 4).
Xue et al. (2020) [45] RCT; China	Cardiac surgery. N = 133: intervention 67, control 66. Age: 58.9 ± 14.2 vs. 57.1 ± 18.0 y; male 39/67 vs. 34/66.	Structured individualized preoperative education using tailored verbal discussion, educational leaflets, demonstrations and ICU/environment familiarization; adapted to age, sex, education and type of surgery.	Routine/unstructured perioperative education.	CAM-ICU first at approximately 12–18 h after surgery, then daily. Primary delirium outcome followed during the first postoperative week.
Hu et al. (2015) [30] RCT; China	Scheduled cardiac surgery/CSICU. 50 randomized; 45 analyzed: intervention 20, control 25. Age: 56.6 ± 11.0 vs. 56.8 ± 11.2 y. Male: 11/20 vs. 16/25.	Earplugs and eye masks during nocturnal sleep plus 30 min relaxing music, in addition to routine care.	Routine postoperative CSICU care.	RCSQ and urinary melatonin/cortisol assessed the night before surgery and first and second postoperative nights. No direct delirium outcome.
Kasapoğlu et al. (2022) [46] Three-arm RCT; Türkiye	General/pulmonary ICU. 107 randomized; 94 completed: Group 1 = 30, Group 2 = 31, control = 33. Age: NR; sex: NR.	Group 1: 10-min non-family orientation recording, daily newspaper and nighttime eye patch. Group 2: identical protocol with family-member voice. Applied for 3 consecutive days.	Standard nursing care.	Delirium presence/absence assessed twice daily for 3 consecutive days.
Esfahanian et al. (2022) [47] RCT; Iran	CABG patients. N = 200, approximately 100/100. Age: intervention 62.2 ± 7.5 vs. control 64.7 ± 7.9 y. Sex: NR.	Relaxing/new-age music 1 h twice daily for 7 postoperative days, delivered through headphones.	Routine postoperative care without study music.	CAM-ICU before and after each music session for 7 d; assessment performed by a blinded researcher.
Kitisin et al. (2025) [48] Single-blind RCT; Thailand	Surgical ICU patients ≥ 65 y. 181 recruited; 177 analyzed: intervention 89, control 88. Sex: NR.	Multicomponent sleep protocol: noise/light reduction, closed doors, alarm adjustment, earplugs, eye masks and limitation of nonessential nighttime procedures. Median intervention exposure 2 nights [IQR 1–3; range 1–7].	Usual care.	Thai CAM-ICU by blinded evaluators for 7 d; RCSQ measured daily.
Zhang et al. (2022) [49] RCT; China	Patients ≥ 65 y undergoing laparoscopic cholecystectomy. 598 randomized; 547 analysed: TEAS 273, control 274. Sex: NR.	Active transcutaneous electrical acupoint stimulation initiated before anesthesia induction and continued until 45 min after surgery.	Sham stimulation at the same acupoints with the electrical output disconnected.	Delirium was a secondary outcome; trained assessors used CAM-ICU with RASS from POD1 through POD3.
Erbay Dallı et al. (2023) [32] Single-blind RCT; Türkiye	Mechanically ventilated ICU patients already delirium-positive. N = 36: music 12, noise-reduction 12, control 12. Age: NR; sex: NR.	Music intervention or environmental noise-reduction intervention twice daily for 5 d.	Routine ICU care.	CAM-ICU/CAM-ICU-7 with additional pain, sedation and anxiety instruments; repeated during the 5-day intervention. This is delirium-management rather than primary-prevention evidence.
Faustino et al. (2022) [50] RCT; Brazil	Critically ill adults in three ICUs. 160 enrolled; 144 analysed: 72/72. Age: NR; sex: NR.	Bundle of periodic reorientation, cognitive stimulation, correction of sensory deficits, environmental management and sleep promotion throughout ICU stay.	Standard ICU care.	CAM-ICU Flowsheet twice daily throughout ICU stay. Primary endpoint: delirium incidence density.
Zhang et al. (2017) [22] Prospective before-after study; China	CABG cardiothoracic ICU. N = 278: intervention 141, historical control 137. Age: 63.09 ± 8.49 vs. 63.45 ± 9.26 y; male 78.0% vs. 80.3%.	Risk-factor-targeted protocol including pain control, early catheter removal, 5W1H orientation, increased family visits, reduction of care interruptions, comfort measures and sleep monitoring.	Historical routine-care cohort.	CAM-ICU; delirium monitored during the first 7 postoperative days.
Chen et al. (2021) [23] Quasi-experimental; China	OPCABG patients in cardiac ICU. N = 67: intervention 30, control 37. Age: NR; sex: NR.	Evidence-based interprofessional sleep-promotion protocol reducing nighttime sound, light and unnecessary nocturnal interventions.	Usual cardiac ICU care.	RCSQ plus environmental measurements during two consecutive postoperative nights. No direct delirium assessment.

Note: RCT = randomized controlled trial; ICU = intensive care unit; CABG = coronary artery bypass grafting; CAM = Confusion Assessment Method; CAM-ICU = Confusion Assessment Method for the Intensive Care Unit; Nu-DESC = Nursing Delirium Screening Scale; DOSS = Delirium Observation Screening Scale; DSM-5 = Diagnostic and Statistical Manual of Mental Disorders, Fifth Edition; DDS = Delirium Detection Score; RASS = Richmond Agitation-Sedation Scale; RCSQ = Richards–Campbell Sleep Questionnaire; 4AT = 4 A’s Test; HELP = Hospital Elder Life Program; TEAS—Transcutaneous Electrical Acupoint Stimulation; OPCABG = off-pump coronary artery bypass grafting; POD = postoperative day. Values are presented as reported by the original studies. Where precise intervention frequency, duration, demographic information, or follow-up was not specified in the primary report, no value was imputed. Hu et al. (2015) [30] and Chen et al. (2021/2022) [23] evaluated postoperative sleep-related outcomes without a direct delirium outcome. Kim et al. (2020) [31] primarily evaluated postoperative sleep and neuroendocrine outcomes but additionally reported postoperative delirium. Erbay Dallı et al. (2023) [32] enrolled patients with established delirium and was therefore considered evidence related to delirium management rather than primary prevention.

### 3.3. Risk of Bias Results

**Figure 2:**
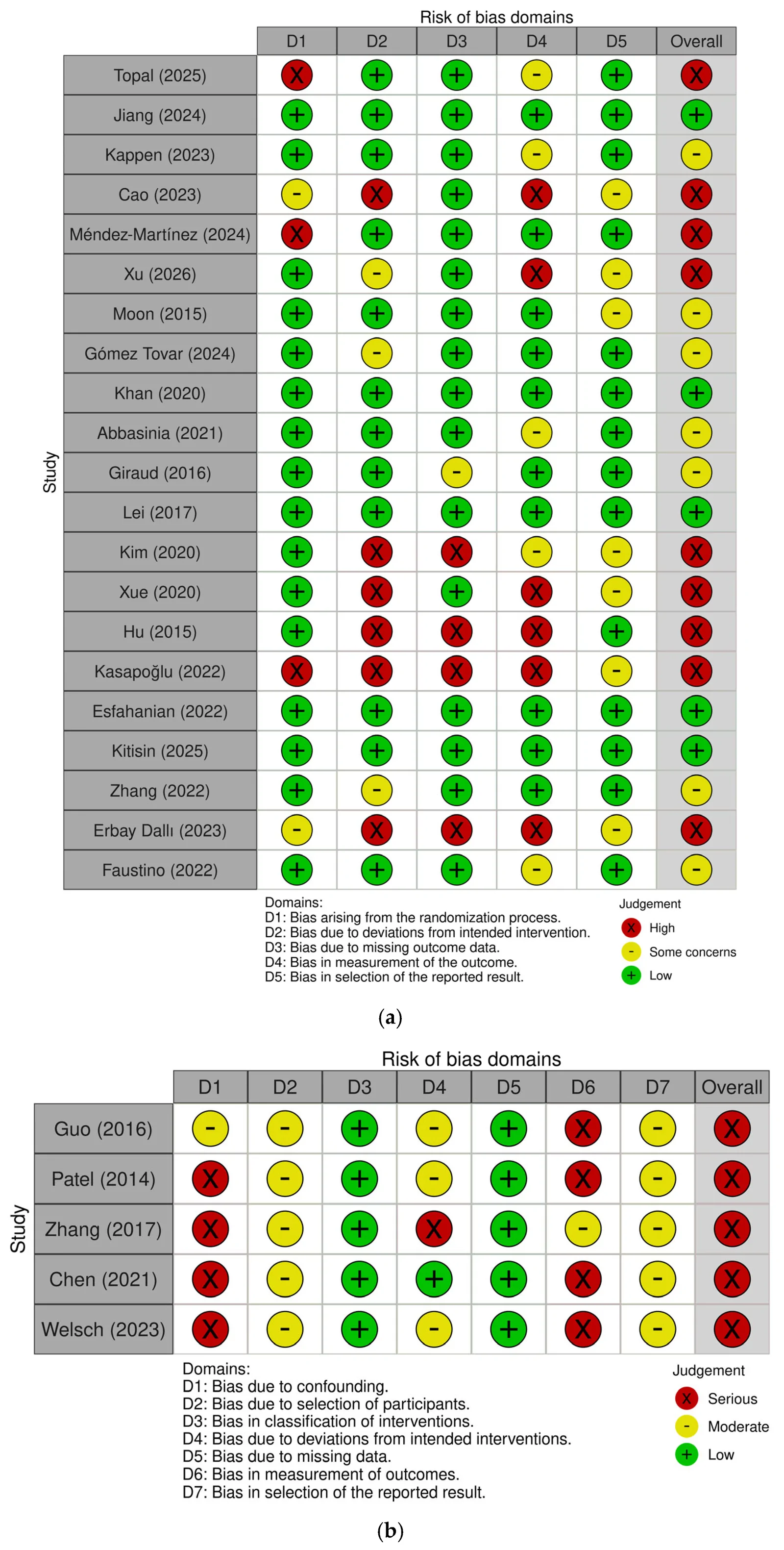
(**a**) Risk-of-bias assessment of the 21 randomized studies using the Cochrane Risk of Bias 2 tool. Visual summary generated using ROBVIS. (**b**) Risk-of-bias assessment of the five non-randomized studies using the ROBINS-I tool. Visual summary generated using ROBVIS. The randomized studies shown in panel (**a**) correspond to references [30,31,32,33,34,35,36,37,38,39,40,41,42,43,44,45,46,47,48,49,50], and the non-randomized studies shown in panel (**b**) correspond to references [19,20,21,22,23].

Risk of bias was assessed according to study design. Of the 26 studies included in the systematic review, 21 randomized studies were evaluated using the Cochrane Risk of Bias 2 tool (RoB 2), whereas five non-randomized studies were assessed using the Risk of Bias in Non-randomized Studies of Interventions tool (ROBINS-I) (Figure 2).

Among the 21 randomized studies assessed with RoB 2, five studies were judged to have a low overall risk of bias, seven presented some concerns, and nine were judged to have a high overall risk of bias. The most frequent methodological concerns were identified in measurement of the outcome (Domain 4), deviations from intended interventions (Domain 2), and selection of the reported result (Domain 5).

In Domain 4, ten studies were judged to have a low risk of bias, five presented some concerns, and six were judged to have a high risk of bias. In Domain 2, 12 studies were judged to have a low risk of bias, three presented some concerns, and six were judged to have a high risk of bias. Domain 5 was judged as low risk in 14 studies and as presenting some concerns in seven studies.

Bias arising from the randomization process (Domain 1) was judged as low in 16 studies, while two studies presented some concerns and three were judged to have a high risk of bias. Bias due to missing outcome data (Domain 3) was generally low, with 16 studies judged as low risk, one presenting some concerns, and four judged to have a high risk of bias.

Five non-randomized studies—Guo et al. (2016) [19], Patel et al. (2014) [20], Welsch et al. (2023) [21], Zhang et al. (2017) [22], and Chen et al. (2021) [23]—were assessed using ROBINS-I. All five studies were judged to have a serious overall risk of bias, although none was considered to have a critical risk of bias. The principal concerns were related to confounding and outcome measurement.

Confounding was judged to be serious in Patel et al. (2014) [20], Welsch et al. (2023) [21], Zhang et al. (2017) [22], and Chen et al. (2021) [23], primarily because of non-random intervention assignment, participant self-selection, non-concurrent comparison groups, inadequate adjustment for important prognostic variables, or susceptibility to temporal changes in clinical practice. Guo et al. (2016) [19] was judged to have a moderate risk of bias due to confounding.

Serious concerns regarding outcome measurement were identified in Guo et al. (2016) [19], Patel et al. (2014) [20], Welsch et al. (2023) [21], and Chen et al. (2021) [23], mainly because outcome assessors were not blinded, outcome measurement involved subjective clinical judgments, or assessment relied on routine clinical documentation. Zhang et al. (2017) [22] was judged to have a moderate risk of bias in this domain.

Overall, the risk-of-bias assessment identified important methodological limitations across both randomized and non-randomized studies. Therefore, pooled effect estimates and findings from the qualitative synthesis should be interpreted cautiously, particularly because several studies were vulnerable to non-blinded outcome assessment, residual confounding, deviations from intended interventions, and selective reporting.

### 3.4. Studies Included in the Primary Meta-Analysis

From the 26 studies included in the systematic review, 14 studies provided sufficient dichotomous data to evaluate the primary outcome, postoperative delirium incidence. These studies reported both the number of patients who developed delirium and the total number of participants in the intervention and control groups. The interventions included music-based strategies, cognitive training, family engagement, multicomponent bundles, mirror intervention, cerebral monitoring, and sleep promotion. Study-level data for the 14 studies included in the primary meta-analysis are summarized in Table 2.

### 3.5. Primary Outcome: Incidence of Delirium

**Figure 3:**
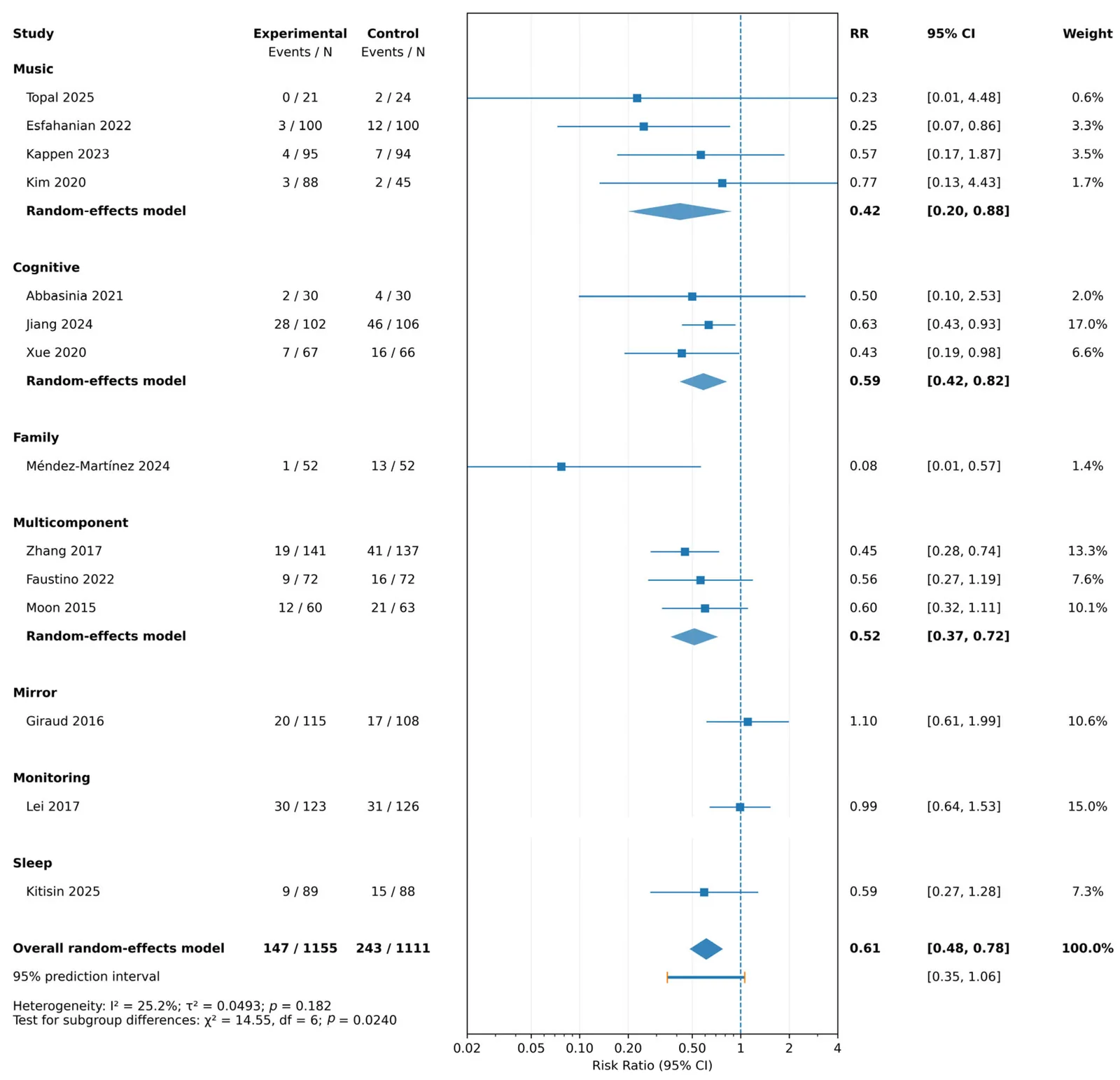
Forest plot of the primary meta-analysis evaluating postoperative delirium incidence. Squares represent individual-study risk ratios and horizontal lines represent the corresponding 95% confidence intervals. Diamonds represent subgroup and overall random-effects pooled estimates. Bold text identifies subgroup and overall pooled estimates. The vertical dashed line indicates the null effect (RR = 1). The studies displayed correspond to references [22,31,33,34,35,37,39,42,43,44,45,47,48,50].

Fourteen studies were included in the revised meta-analysis of postoperative delirium incidence, comprising 1155 participants allocated to non-pharmacological interventions and 1111 participants allocated to comparator conditions. A total of 147 delirium events occurred among intervention participants and 243 among controls. The random-effects model yielded a pooled risk ratio of RR = 0.61 (95% CI: 0.48–0.78), indicating a significantly lower incidence of delirium among patients receiving non-pharmacological interventions. Statistical heterogeneity was low to moderate (I^2^ = 25.2%; τ^2^ = 0.0493; *p* = 0.182). The revised outcome selection and study-level data did not materially alter the magnitude or direction of the primary pooled effect. The forest plot of the primary meta-analysis is presented in Figure 3.

Subgroup analyses according to intervention category continued to show variation in the magnitude and precision of the pooled effect estimates. The test for subgroup differences remained statistically significant (χ^2^ = 14.55, df = 6, *p* = 0.0240), indicating that effect estimates differed across intervention categories. Family-based interventions showed the largest estimated effect, although this subgroup was represented by only one study in the revised incidence meta-analysis. Multicomponent, cognitive, and music-based interventions also favored non-pharmacological prevention, whereas isolated cerebral monitoring and mirror interventions showed estimates closer to the null. Given the small number of studies within several categories, these subgroup findings should be interpreted as exploratory rather than as definitive evidence of differential effectiveness between intervention types.

The 95% prediction interval for the overall random-effects estimate was 0.35–1.06, which included the null value (1.0). These findings indicate variability in the effects across the included studies and intervention categories.

### 3.6. Publication Bias Assessment

**Figure 4:**
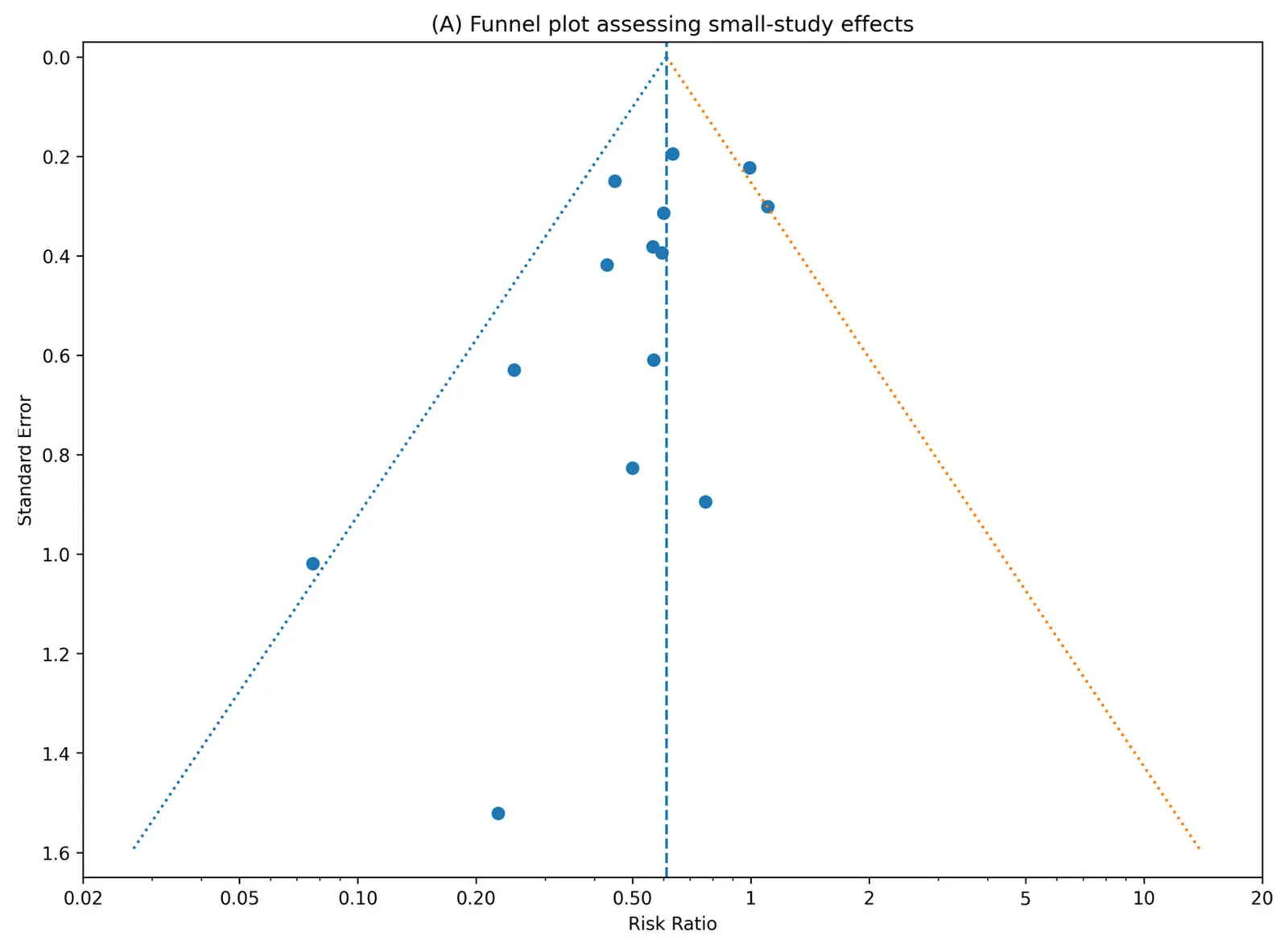

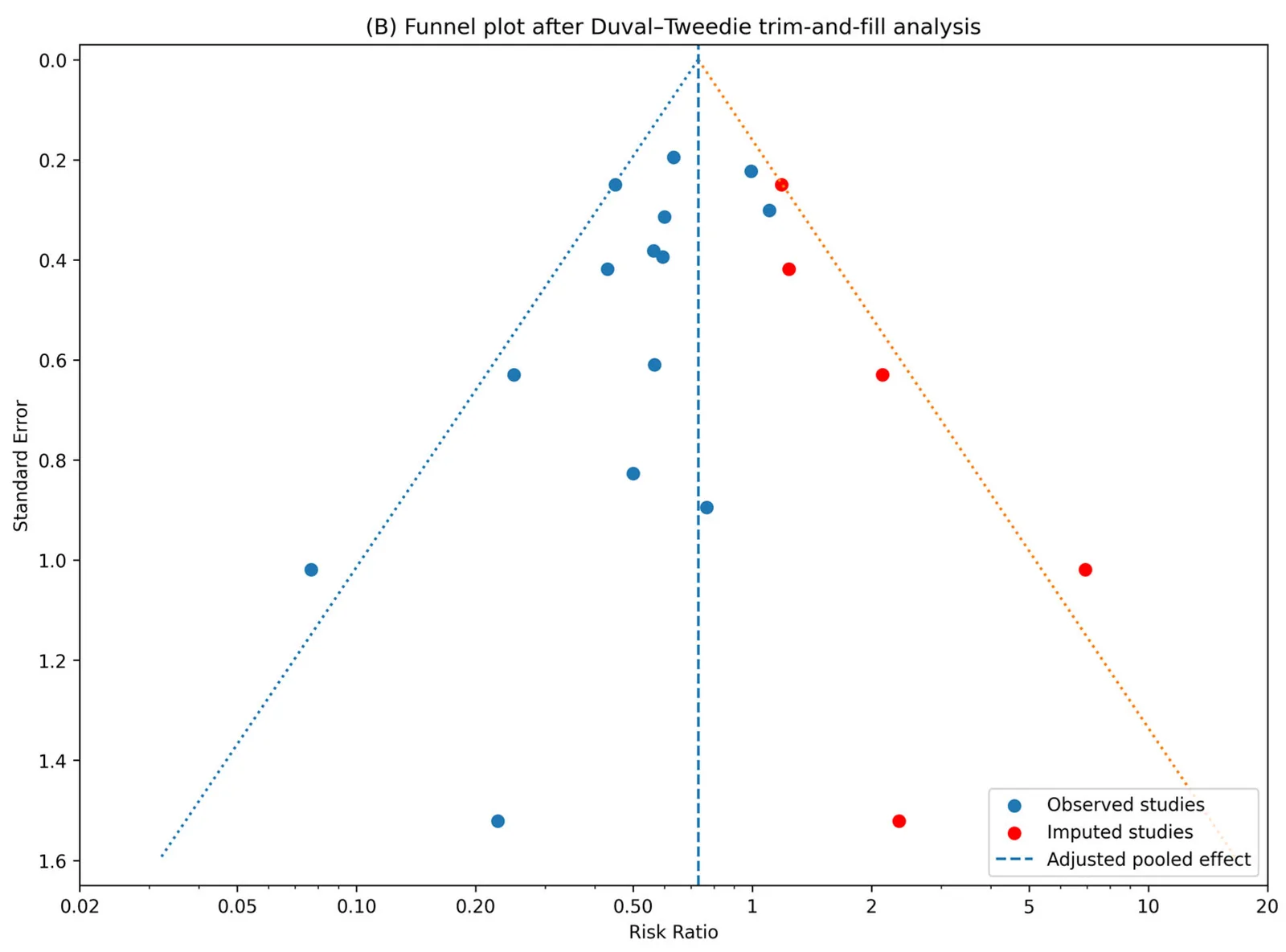
(**A**) Funnel plot assessing small-study effects among the 14 studies included in the meta-analysis of postoperative delirium incidence. (**B**) Funnel plot after the exploratory Duval–Tweedie trim-and-fill analysis. Blue filled circles represent the 14 observed studies, whereas red filled circles represent the five hypothetical imputed studies on the less favorable side of the funnel plot. The blue and orange dotted diagonal lines indicate the approximate funnel limits. The blue vertical dashed line in panel (**A**) indicates the observed pooled random-effects estimate, whereas the blue vertical dashed line in panel (**B**) indicates the adjusted pooled random-effects estimate after trim-and-fill (RR = 0.73; 95% CI: 0.57–0.93).

Potential small-study effects were assessed through visual inspection of the funnel plot and Egger’s regression test. The revised funnel plot showed some asymmetry among smaller studies. Egger’s regression test yielded an intercept of −1.18 (SE = 0.59; t = −2.01; df = 12; *p* = 0.068). Using the two-sided *p* < 0.10 interpretive threshold adopted during peer review for this exploratory assessment, the result remained suggestive of possible small-study effects. Funnel-plot asymmetry was not interpreted as definitive evidence of publication bias because asymmetry may also arise from clinical heterogeneity, methodological differences, and small-study effects unrelated to selective publication. The corresponding funnel plots are presented in Figure 4.

An exploratory Duval–Tweedie trim-and-fill analysis estimated five potentially missing studies on the side of the funnel plot associated with less favorable intervention effects. After imputation, the adjusted pooled estimate was attenuated to RR = 0.73 (95% CI: 0.57–0.93), but remained statistically significant and favored non-pharmacological interventions. The trim-and-fill analysis was therefore considered a sensitivity analysis rather than a corrected estimate of the true intervention effect.

### 3.7. Sensitivity Analysis

A leave-one-out sensitivity analysis was performed by sequentially removing each of the 14 studies from the revised primary meta-analysis and recalculating the pooled effect using the same random-effects model. The pooled risk ratio remained stable across all iterations, ranging from RR = 0.57 to RR = 0.65, and all 95% confidence intervals excluded the null effect. No individual study materially altered the magnitude or direction of the pooled association, indicating that the primary result was not driven by a single study (Supplementary Table S5, Panel A).

A sensitivity analysis restricted to randomized controlled trials excluded Zhang et al. (2017) [22], the only non-randomized study contributing to the revised primary meta-analysis. Thirteen randomized controlled trials comprising 1988 participants remained. The pooled estimate was RR = 0.65 (95% CI: 0.51–0.83), with low-to-moderate heterogeneity (I^2^ = 20.9%; τ^2^ = 0.0366). The direction and statistical significance of the effect remained consistent with the primary analysis. (Supplementary Table S5, Panel B).

The prespecified sensitivity analysis according to methodological quality excluded randomized trials judged to have high overall risk of bias and the non-randomized study judged to have serious overall risk of bias. Nine randomized trials classified as low risk or some concerns remained, comprising 1573 participants. The pooled effect remained statistically significant (RR = 0.70; 95% CI: 0.55–0.89), with low heterogeneity (I^2^ = 7.9%; τ^2^ = 0.0190).

Exploratory stratification of randomized trials according to overall RoB 2 classification yielded pooled RRs of 0.68 (95% CI: 0.46–0.99) for four low-risk trials, 0.71 (95% CI: 0.49–1.04) for five trials with some concerns, and 0.37 (95% CI: 0.19–0.73) for four high-risk trials. The larger estimated effect among high-risk studies suggests that methodological limitations may have influenced the magnitude of the pooled association. Nevertheless, exclusion of studies at high or serious overall risk of bias did not eliminate the statistically significant preventive association.

The primary meta-analysis was additionally repeated using the Hartung–Knapp adjustment while retaining REML estimation of between-study variance. The pooled point estimate remained RR = 0.61, with a 95% confidence interval of 0.47–0.79, and therefore the statistical interpretation of the primary analysis was unchanged.

Because the final review included a small number of broader critically ill ICU populations in addition to clearly postoperative or surgical ICU cohorts, a post hoc sensitivity analysis was also performed restricting the synthesis to studies conducted in clearly postoperative or surgical populations. Twelve studies comprising 1999 participants remained, producing a consistent effect (RR = 0.60; 95% CI: 0.45–0.81; I^2^ = 36.2%; τ^2^ = 0.0839). This analysis indicated that the overall preventive association was not dependent on the inclusion of broader mixed-ICU populations.

### 3.8. Certainty of the Evidence

Fourteen studies contributed to the revised primary pooled estimate, including 13 randomized controlled trials and one non-randomized prospective before–after study. The certainty of the evidence for postoperative delirium incidence was assessed using the GRADE approach. The starting certainty was considered high because the evidence base was predominantly randomized. The certainty was downgraded by one level for risk of bias because four randomized trials contributing to the primary analysis were judged to have high overall risk of bias and the non-randomized study was judged to have serious overall risk of bias using ROBINS-I.

No downgrading was applied for inconsistency because statistical heterogeneity remained low to moderate (I^2^ = 25.2%; τ^2^ = 0.0493), and the overall direction of effect generally favored intervention. Indirectness was not considered serious because most contributing populations were postoperative or surgical ICU populations, and a sensitivity analysis restricted to clearly postoperative or surgical populations produced a consistent effect (RR = 0.60; 95% CI: 0.45–0.81). No downgrading was applied for imprecision because the primary random-effects estimate was RR = 0.61 (95% CI: 0.48–0.78) and its confidence interval excluded the null effect. Using an assumed baseline risk of 219 per 1000 participants, the corresponding intervention risk was 134 per 1000 (95% CI: 105–170), corresponding to approximately 85 fewer delirium events per 1000 patients (95% CI: 49–113 fewer).

Egger’s regression test remained suggestive of possible small-study effects at the *p* < 0.10 threshold (*p* = 0.068). The exploratory trim-and-fill analysis estimated five potentially missing studies and attenuated the pooled estimate to RR = 0.73 (95% CI: 0.57–0.93), without changing the direction or statistical significance of the association. Because funnel-plot asymmetry is not specific to publication bias and the adjusted estimate remained statistically significant, no additional downgrade was applied for publication bias. The overall certainty of the evidence was therefore rated as moderate. The sensitivity analysis restricted to the 13 randomized controlled trials yielded a consistent effect (RR = 0.65; 95% CI: 0.51–0.83). Detailed judgments are presented in Supplementary Table S6. A Summary of Findings for the primary outcome is presented in Table 3.

### 3.9. Secondary Outcome Results

Exploratory secondary analyses showed no statistically significant effects on delirium duration (k = 3; MD = −1.18 days; 95% CI: −3.38 to 1.03; I^2^ = 83.5%), delirium severity (k = 3; SMD = −0.56; 95% CI: −2.40 to 1.29; I^2^ = 82.7%) or ICU length of stay (k = 4; MD = −0.18 days; 95% CI: −0.65 to 0.30; I^2^ = 24.4%). Detailed results of the exploratory secondary outcome meta-analyses are presented in Supplementary Table S2.

## 4. Discussion

### 4.1. Principal Findings and Interpretation of Postoperative Delirium Incidence

The primary outcome of this systematic review was delirium incidence among critically ill adult patients receiving non-pharmacological preventive interventions. Twenty-six studies were included in the qualitative synthesis, and 14 studies—13 randomized controlled trials and one non-randomized prospective before–after study—provided methodologically compatible dichotomous incidence data for the revised primary meta-analysis. The pooled random-effects estimate was RR = 0.61 (95% CI: 0.48–0.78), corresponding to an approximate 39% relative reduction in delirium risk. A sensitivity analysis restricted to the 13 randomized controlled trials produced a consistent estimate (RR = 0.65; 95% CI: 0.51–0.83), and a separate analysis restricted to clearly postoperative or surgical populations also remained statistically significant (RR = 0.60; 95% CI: 0.45–0.81). These findings indicate that the overall association was not dependent on either the non-randomized study or the broader mixed-ICU populations.

This finding is clinically relevant because postoperative delirium is a frequent complication in critically ill surgical patients and is associated with worse clinical outcomes, including prolonged hospitalization, functional decline, increased healthcare burden, and greater patient and family distress [1,2]. Therefore, reducing the incidence of delirium is important not only as a statistical finding, but also as a meaningful clinical outcome for postoperative ICU care.

The lower incidence observed in the intervention groups is also clinically plausible. Postoperative delirium is a multifactorial syndrome that results from the interaction between baseline patient vulnerability and modifiable precipitating factors. In the ICU, postoperative patients are exposed to several delirium triggers, including pain, sleep disruption, sensory deprivation, immobility, environmental stress, anxiety, sedative exposure, mechanical ventilation, and separation from family members. Non-pharmacological interventions may decrease the incidence of delirium because they target several of these precipitating factors simultaneously [4,8,10,11].

From this perspective, the reduction in delirium incidence should not be interpreted as the effect of a single isolated mechanism. Rather, it likely reflects the combined contribution of multiple preventive actions. Cognitive stimulation and reorientation may support attention and awareness; sleep-promotion strategies may help preserve circadian rhythm; sensory optimization may reduce perceptual misinterpretation; early mobilization may reduce immobility-related vulnerability; music may decrease anxiety, pain, stress responses, and sedative exposure; and family engagement may provide familiar cues, emotional security, and orientation support [4,5,8,12]. Together, these mechanisms may explain why non-pharmacological interventions were associated with fewer new cases of postoperative delirium.

The low-to-moderate heterogeneity observed in the primary meta-analysis (I^2^ = 25.2%) also strengthens the interpretation of this finding. Although the included studies differed in intervention type, surgical population, ICU context, and delirium assessment tools, the overall direction of the effect favored non-pharmacological interventions. This suggests that the reduction in delirium incidence was reasonably consistent across different clinical scenarios.

### 4.2. Differences According to Intervention Type

The subgroup analysis suggests that the reduction in postoperative delirium incidence varied according to the type of non-pharmacological intervention. Some strategies appeared to be associated with a greater decrease in incident delirium than others. In particular, family-based interventions showed one of the most favorable subgroup effects, followed by multicomponent strategies, cognitive interventions, and music-based interventions. In contrast, isolated monitoring strategies and mirror-based interventions showed less consistent effects on delirium incidence.

Importantly, the pooled estimate should not be interpreted as implying that all non-pharmacological interventions are clinically equivalent or act through a single biological mechanism. Rather, these interventions constitute a clinically related class of preventive strategies addressing different modifiable precipitating factors in the multifactorial pathogenesis of delirium, including sleep–wake disruption, disorientation, sensory deprivation, immobility, anxiety, environmental stress, family separation, and sedation-related factors. The pooled random-effects estimate therefore represents an average preventive effect across heterogeneous strategies. The significant subgroup-difference test and the prediction interval further indicate that the magnitude of benefit may vary according to intervention type and clinical context.

Family-based interventions may be especially relevant because they address emotional, cognitive, and environmental dimensions of delirium prevention. In the postoperative ICU setting, patients are frequently disoriented, anxious, exposed to unfamiliar stimuli, and separated from their usual sources of emotional support. Family presence or family-related stimuli may help reduce the incidence of delirium by providing familiar voices, autobiographical cues, reassurance, emotional containment, and orientation to person, place, and time. This may explain why family-visit nursing care, family-image projection, and family presence were clinically plausible strategies for reducing new delirium episodes [21,36,37].

This finding is consistent with the ABCDEF bundle, where family engagement and empowerment are considered essential components of ICU care and delirium prevention [5]. In this sense, family participation should not be viewed only as a humanizing element of care, but also as a potentially protective intervention against postoperative acute brain dysfunction. However, this interpretation should be considered cautiously because the number of family-based studies was limited, and some trials had small sample sizes.

Multicomponent interventions also showed a favorable effect on delirium incidence. This is coherent with the multifactorial nature of delirium, since these interventions usually combine reorientation, sleep promotion, sensory support, pain control, environmental management, early mobilization, and family involvement. Because postoperative delirium does not usually arise from a single cause, preventive strategies that simultaneously address several modifiable risk factors may be more effective than isolated measures [6,7,8,20,39,40,42,46,50].

Music-based interventions were among the most frequently evaluated strategies in the included studies. Among studies assessing delirium prevention, their potential effect on incident delirium may be related to reductions in anxiety, pain, stress responses, agitation, and sedative exposure, all of which contribute to delirium vulnerability in critically ill patients. However, not all music-based studies evaluated primary prevention. Hu et al. (2015) [30] primarily assessed postoperative sleep-related outcomes without directly measuring delirium incidence. Kim et al. (2020) [31] primarily investigated postoperative sleep quality and neuroendocrine outcomes but additionally assessed postoperative delirium during the ICU stay. Erbay Dallı et al. (2023) [32] included patients with established delirium and evaluated changes in delirium severity and related clinical outcomes. Therefore, these studies should be interpreted as indirect supportive or delirium-management evidence rather than direct evidence of primary prevention. Music should not be regarded as a stand-alone preventive strategy but as a potentially supportive component of broader non-pharmacological programs.

Sleep-promotion interventions target modifiable factors associated with delirium vulnerability, including sleep disruption, excessive noise, continuous light exposure, frequent nocturnal interruptions, and circadian rhythm alteration. Interventions such as eye masks, earplugs, environmental modification, reduction of light and noise, music, and structured sleep protocols may improve postoperative sleep quality and preserve circadian organization. However, improved sleep quality should not automatically be interpreted as a reduction in delirium incidence. Hu et al. (2015) [30] and Chen et al. (2021) [23] did not directly assess incident delirium and therefore provided indirect supportive evidence only. Kim et al. (2020) [31], although primarily designed to evaluate postoperative sleep quality, additionally assessed postoperative delirium during the ICU stay. Accordingly, sleep-related improvements should not automatically be interpreted as evidence of delirium prevention, and conclusions regarding delirium should be based specifically on the delirium outcomes reported by each study. Direct preventive conclusions should be based on studies that measured delirium using validated diagnostic or screening instruments.

### 4.3. Interpretation of Secondary Outcomes

The exploratory analyses of secondary outcomes did not demonstrate statistically significant effects of non-pharmacological interventions on delirium duration, delirium severity, or ICU length of stay. Although the point estimate for delirium duration suggested a reduction of 1.18 days, the confidence interval included the null value (MD = −1.18 days; 95% CI: −3.38 to 1.03). Similarly, the estimated effect on delirium severity favored the intervention (SMD = −0.56), but the confidence interval was wide and included the null value (95% CI: −2.40 to 1.29). For ICU length of stay, the pooled estimate was close to the null value (MD = −0.18 days; 95% CI: −0.65 to 0.30).

The interpretation of these findings should take into account the limited body of available evidence. Both delirium duration and severity were assessed in only three studies and showed substantial heterogeneity (I^2^ = 83.5% and 82.7%, respectively). This variability may reflect clinical and methodological differences across the contributing studies, including variation in the characteristics and intensity of the non-pharmacological interventions, patient populations, and delirium assessment procedures. Differences in the timing and frequency of assessments, intervention exposure, and follow-up periods may have further contributed to the observed variability. Given the small number of contributing studies, these potential sources of heterogeneity could not be reliably explored through formal subgroup or meta-regression analyses.

In contrast, the analysis of ICU length of stay included four studies and showed low heterogeneity (I^2^ = 24.4%), suggesting greater consistency across individual study estimates. Nevertheless, this consistency did not translate into evidence of an intervention effect, as the confidence interval included both a potential reduction and a potential increase in ICU length of stay. Thus, the absence of a statistically significant effect for this outcome appears relatively consistent across studies, although it does not establish the absence of a potential effect.

### 4.4. Clinical Implications

The main clinical implication of this review is that non-pharmacological interventions may reduce the incidence of postoperative delirium in critically ill adult patients. This is relevant because the primary outcome of the meta-analysis was not only statistically significant, but also clinically meaningful: fewer patients developed delirium when exposed to structured non-pharmacological preventive strategies compared with standard care.

In postoperative ICU practice, reducing the incidence of delirium should be considered a priority because delirium is associated with worse outcomes, increased care complexity, prolonged recovery, distress for patients and families, and greater healthcare burden [1,2]. Therefore, interventions that reduce the occurrence of new delirium episodes may contribute to safer, more humanized, and higher-quality postoperative critical care.

The findings support the integration of non-pharmacological delirium-prevention strategies into routine ICU care. Many individual non-pharmacological strategies are low-risk and may require limited material resources; however, the feasibility of implementing structured multicomponent programs depends on local staffing, workflow, training, organizational support, and available resources. Unlike pharmacological prevention strategies, which may expose patients to medication-related complications and have shown inconsistent preventive effects, non-pharmacological approaches can be applied as part of daily interdisciplinary care [4,6,8].

Nursing teams have a central role in reducing the incidence of postoperative delirium because many of the effective interventions identified in this review are directly connected to nursing practice. These include patient reorientation, sleep hygiene, sensory optimization, environmental control, family engagement, early stimulation, pain control, mobilization support, and adherence to structured delirium-prevention protocols [7,8,19,20,22,39,40,42,46,50]. For this reason, delirium prevention should not be understood as an isolated intervention, but as a continuous process embedded in postoperative monitoring, communication, patient safety, and humanized critical care.

The subgroup findings also suggest that family engagement should receive particular attention in postoperative ICU settings. Family-based interventions may help reduce the incidence of delirium by providing emotional security, familiar stimuli, reassurance, and orientation support. In this sense, family participation should be considered not only a psychosocial component of care, but also a potentially protective strategy against acute brain dysfunction [5,21,36,37].

These results also support the implementation of multicomponent care bundles. Because postoperative delirium is multifactorial, isolated interventions may be insufficient for many patients. Structured bundles that combine reorientation, sleep promotion, sensory optimization, early mobilization, environmental management, pain control, and family involvement may be more appropriate for high-risk postoperative ICU patients, including older adults, cardiac surgery patients, mechanically ventilated patients, and patients with baseline vulnerability [4,5,6,7,8,20,39,40,42,46,50]. The translation of these multicomponent approaches into routine practice nevertheless requires consideration of implementation conditions. Protocol effectiveness may depend on adequate nurse-to-patient staffing, workload, staff education and training, interdisciplinary coordination, leadership support, and sustained adherence to individual bundle components. Interventions involving repeated reorientation, mobilization, sleep protection, environmental modification, family participation, and structured delirium assessment require time and coordination and may be more difficult to deliver consistently during periods of high workload or limited staffing. Likewise, feasibility may vary according to ICU infrastructure, visiting policies, availability of rehabilitation personnel, and access to sensory or monitoring resources. Therefore, the efficacy observed under study conditions should not be assumed to translate automatically into equivalent effectiveness in every clinical setting. Implementation strategies should be adapted to local resources, and future trials should report intervention fidelity, protocol adherence, staffing requirements, nursing workload, and barriers and facilitators to implementation.

From a health systems perspective, reducing the incidence of postoperative delirium may contribute to better quality of care and more efficient use of critical care resources. Even if ICU length of stay was not significantly reduced in the secondary analysis, preventing new delirium episodes remains clinically valuable because it may reduce patient distress, improve recovery trajectories, decrease the burden of acute brain dysfunction, and support safer postoperative care.

### 4.5. Strengths and Limitations

This review has several strengths. First, it was conducted and reported according to PRISMA 2020 recommendations and used a broad search strategy across multiple databases. Second, it incorporated evidence from different countries, surgical populations, critical care settings, and categories of non-pharmacological interventions. Third, the primary outcome was clearly defined as postoperative delirium incidence and was synthesized using a prespecified random-effects model. Fourth, the revised primary meta-analysis included 14 studies and 2266 participants, and statistical heterogeneity was low to moderate. The leave-one-out analysis and the sensitivity analysis restricted to randomized controlled trials produced consistent results, supporting the robustness of the primary finding.

Several limitations should also be acknowledged. First, the included studies were clinically heterogeneous regarding surgical populations, ICU contexts, intervention components, intensity and duration of exposure, and comparator conditions. Second, delirium was assessed using different instruments, thresholds, and assessment schedules, potentially affecting outcome classification. Third, several randomized studies presented some concerns or high risk of bias, and the non-randomized studies presented serious risk of bias, particularly because of residual confounding, lack of blinding, deviations from intended interventions, and outcome measurement. The additional risk-of-bias sensitivity analysis provides further context for this limitation. Excluding studies with high or serious overall risk of bias attenuated the pooled estimate from RR 0.61 to RR 0.70 but did not eliminate the statistically significant preventive association. Exploratory analyses also showed substantially larger estimated effects among the four high-risk randomized trials than among trials classified as low risk or some concerns. These findings suggest that methodological limitations may have inflated the magnitude of the overall effect, while the persistence of the association among studies with more favourable methodological assessments supports the robustness of the direction of effect.

Fourth, only 14 of the 26 included studies provided comparable dichotomous data for the primary meta-analysis, and one of these 14 studies used non-randomized designs. Although the sensitivity analysis restricted to 13 randomized controlled trials produced a consistent effect, the primary pooled estimate should still be interpreted in light of the methodological limitations of the contributing studies. The exclusion of 12 studies from the primary quantitative synthesis may have limited the breadth of evidence contributing to the pooled effect estimate. However, these studies were not omitted on the basis of the direction or statistical significance of their results; rather, they did not provide comparable dichotomous data on incident postoperative delirium or evaluated supportive, non-comparable, or management-related outcomes. They were therefore retained in the qualitative synthesis. Although this approach reduces the risk of outcome-driven study selection, the possibility of availability bias cannot be completely excluded, and the pooled estimate should be interpreted as representing the subset of studies that provided sufficiently comparable incidence data rather than the entire qualitative evidence base.

Fifth, two studies—Hu et al. (2015) [30] and Chen et al. (2021) [23]—assessed postoperative sleep-related outcomes without directly measuring incident delirium, while one study evaluated the management of established delirium. These studies contributed only indirect or complementary qualitative evidence and should not be interpreted as demonstrating primary prevention.

Implementation-related outcomes, including staffing requirements, nursing workload, protocol adherence, and intervention fidelity, were not consistently reported across the included studies and therefore could not be quantitatively synthesized. This limits conclusions regarding the real-world feasibility and sustainability of multicomponent programs across different ICU settings.

Sixth, the secondary outcome analyses included only three or four studies and, for delirium duration and severity, showed substantial heterogeneity. These results should therefore be considered exploratory. The restriction to English- and Spanish-language publications may have introduced language bias and could have excluded relevant evidence published in other languages. Eligibility also required a sufficiently complete report to permit assessment of study design, outcomes, and risk of bias. Nevertheless, all 108 reports that reached the full-text retrieval stage were successfully obtained; therefore, no report reaching full-text eligibility assessment was excluded because the full text could not be retrieved. Egger’s regression test remained suggestive of possible small-study effects when applying the *p* < 0.10 threshold (*p* = 0.068). The exploratory trim-and-fill analysis estimated five potentially missing studies and attenuated the pooled estimate from RR = 0.61 to RR = 0.73 (95% CI: 0.57–0.93), without changing the direction or statistical significance of the association. Nevertheless, funnel-plot asymmetry is not specific to publication bias, and the potential influence of small-study effects should be considered when interpreting the magnitude of the pooled estimate.

## 5. Conclusions

This systematic review and meta-analysis found that non-pharmacological interventions were associated with a significant reduction in delirium incidence among critically ill adult patients. The revised primary random-effects analysis of 14 studies and 2266 participants yielded an RR of 0.61 (95% CI: 0.48–0.78). The sensitivity analysis restricted to 13 randomized controlled trials produced a consistent effect (RR = 0.65; 95% CI: 0.51–0.83), as did the analysis restricted to clearly postoperative or surgical populations (RR = 0.60; 95% CI: 0.45–0.81). The certainty of the evidence was rated as moderate, primarily because of methodological limitations in several contributing studies.

Overall, these findings support the potential role of non-pharmacological strategies in postoperative delirium prevention. However, the pooled estimate represents an average effect across different intervention categories and clinical settings and should not be interpreted as evidence of equal effectiveness across individual interventions. The significant subgroup differences and the prediction interval of 0.35–1.06 suggest that effects may vary according to intervention type and context. Further adequately powered randomized controlled trials are needed to determine the effectiveness of specific intervention components and identify the most effective strategies for different postoperative ICU populations.

## Figures and Tables

**Table 2:** Studies included in the revised primary meta-analysis of postoperative delirium incidence (*n* = 14).

No.	First Author (Year)	Country	Surgical Population/Setting	Intervention Category	Delirium Assessment Tool/Outcome Selected	Events/Total EG	Events/Total CG
1	Topal (2025) [33]	Türkiye	Abdominal surgery ICU patients	Music + eye mask	Nu-DESC; postoperative delirium incidence during the 3-day assessment period	0/21	2/24
2	Esfahanian (2022) [47]	Iran	CABG patients	Music therapy	CAM-ICU; cumulative postoperative delirium incidence during 7-day follow-up	3/100	12/100
3	Kappen (2023) [35]	Netherlands	Craniotomy/neurosurgical patients	Perioperative music	DOSS screening followed by DSM-5 confirmation; DSM-5-confirmed delirium selected	4/95	7/94
4	Abbasinia (2021) [42]	Iran	CABG patients in cardiac surgery ICU	Tailored cognitive/educational intervention	CAM-ICU; POD3 selected because cumulative incidence was not reported	2/30	4/30
5	Jiang (2024) [34]	China	CABG patients	Cognitive training	CAM/CAM-ICU; cumulative delirium incidence during POD1–7	28/102	46/106
6	Méndez-Martínez (2024) [37]	Spain	Cardiac surgery patients	Family visual projection	CAM-ICU; postoperative delirium incidence	1/52	13/52
7	Kim (2020) [31]	South Korea	Elderly postoperative ICU patients	Music-based intervention	ICDSC-defined postoperative delirium; interactive music and passive music arms combined	3/88	2/45
8	Xue (2020) [45]	China	Cardiac surgery patients	Individualized preoperative education	CAM-ICU; cumulative postoperative delirium incidence during first postoperative week	7/67	16/66
9	Zhang (2017) [22]	China	CABG patients	Multicomponent nursing protocol	CAM-ICU; postoperative delirium incidence during first 7 postoperative days	19/141	41/137
10	Faustino (2022) [50]	Brazil	ICU patients	Multicomponent non-pharmacological bundle	CAM-ICU Flowsheet; cumulative delirium incidence selected	9/72	16/72
11	Moon (2015) [39]	South Korea	ICU patients	Tailored multicomponent delirium-prevention protocol	CAM-ICU; delirium incidence during ICU follow-up	12/60	21/63
12	Giraud (2016) [43]	United Kingdom	Cardiac surgical ICU patients aged ≥70 years	Mirror intervention	CAM-ICU; delirium incidence during ICU stay	20/115	17/108
13	Lei (2017) [44]	Canada	Cardiac surgery patients	Cerebral oximetry monitoring	CAM-ICU in ICU/CAM after ICU discharge; cumulative postoperative delirium incidence	30/123	31/126
14	Kitisin (2025) [48]	Thailand	Elderly surgical ICU patients	Sleep enhancement protocol	Thai CAM-ICU; cumulative delirium incidence during 7-day follow-up	9/89	15/88

Note: EG = experimental group; CG = control group; ICU = intensive care unit; CABG = coronary artery bypass grafting; CAM = Confusion Assessment Method; CAM-ICU = Confusion Assessment Method for the Intensive Care Unit; Nu-DESC = Nursing Delirium Screening Scale; DOSS = Delirium Observation Screening Scale; DSM-5 = Diagnostic and Statistical Manual of Mental Disorders, Fifth Edition; ICDSC = Intensive Care Delirium Screening Checklist; POD = postoperative day. One independent delirium-incidence estimate was retained per study according to the outcome-selection hierarchy described in Section 2.8. For Kappen et al. (2023) [35], DSM-5-confirmed delirium was selected rather than DOSS screening positivity. For Abbasinia et al. (2021) [42], cumulative incidence was not reported; therefore, the latest reported postoperative assessment (POD3) was selected. For Kim et al. (2020) [31], the two eligible music-intervention groups were combined to provide a single intervention group and avoid double-counting the shared control group. Khan et al. (2020) [41] was excluded from the primary incidence meta-analysis because the published report did not provide an extractable dichotomous delirium-incidence outcome. Welsch et al. (2023) [21] was excluded because new incident delirium could not be distinguished from delirium or confusion already present at baseline. Both studies were retained in the qualitative synthesis.

**Table 3:** Non-pharmacological interventions compared with usual care for preventing postoperative delirium in critically ill adult patients. Summary of Findings.

Outcome	Risk with Usual Care	Risk with Intervention	Relative Effect (95% CI)	Participants (Studies)	Certainty of the Evidence (GRADE)
Incidence of postoperative delirium	219 per 1000	134 per 1000 (105 to 170)	RR 0.61 (0.48 to 0.78)	2266 (14 studies: 13 RCTs and 1 non-randomized study)	⊕⊕⊕◯ MODERATE

Note: The assumed risk with usual care was calculated from the aggregate control event rate across the 14 studies (243/1111). The corresponding intervention risk was calculated using the random-effects pooled risk ratio. This corresponds to approximately 85 fewer delirium events per 1000 patients (95% CI: 49 to 113 fewer). The certainty of the evidence was downgraded by one level for risk of bias. The sensitivity analysis restricted to 13 randomized controlled trials yielded a consistent effect (RR = 0.65; 95% CI: 0.51–0.83). RR, risk ratio; CI, confidence interval; RCT, randomized controlled trial. GRADE certainty ratings: ⊕⊕⊕⊕ = high; ⊕⊕⊕◯ = moderate; ⊕⊕◯◯ = low; ⊕◯◯◯ = very low.

## Data Availability

The data supporting the findings of this systematic review are available from the studies included in the review. Extracted data used for the meta-analyses are available from the corresponding author upon reasonable request.

## Supplementary Materials

The following supporting information can be downloaded at: https://www.mdpi.com/article/10.3390/nursrep16090328/s1, Table S1: Search strategies by database; Table S2: Meta-analyses of secondary outcomes associated with non-pharmacological interventions for postoperative delirium prevention; Table S3: PRISMA 2020 checklist indicating where each reporting item is addressed in the manuscript; Table S4: Reports excluded after full-text assessment and primary reasons for exclusion; Table S5: Sensitivity analyses of postoperative delirium incidence; Table S6: GRADE evidence profile for postoperative delirium incidence; Table S7: Comparison between prospectively registered PROSPERO Version 1.0 (19 February 2026) and the final review methods and protocol amendments.

## Public Involvement Statement

No public involvement in any aspect of this research.

## Guidelines and Standards Statement

This manuscript was prepared and reported in accordance with the Preferred Reporting Items for Systematic Reviews and Meta-Analyses (PRISMA) 2020 Statement [13]. The completed PRISMA 2020 checklist is provided in the Supplementary Materials.

## Use of Artificial Intelligence

During the preparation and revision of this manuscript, the authors used ChatGPT (OpenAI, GPT−5.6) as an AI-assisted tool to support language editing, clarity and organization of the text, drafting assistance, and checking of methodological and statistical descriptions. All AI-assisted outputs were critically reviewed, verified, and revised by the authors. All methodological decisions, data extraction, statistical analyses, interpretation of results, and final content remain the responsibility of the authors.

## Abbreviations

The following abbreviations are used in this manuscript:

**Abbreviation**	**Meaning**
ABCDEF	Assess, Prevent, and Manage Pain; Both Spontaneous Awakening Trials and Spontaneous Breathing Trials; Choice of Analgesia and Sedation; Delirium Assessment, Prevention, and Management; Early Mobility and Exercise; Family Engagement and Empowerment
CABG	Coronary Artery Bypass Grafting
CAM	Confusion Assessment Method
CAM-ICU	Confusion Assessment Method for the Intensive Care Unit
CG	Control Group
CI	Confidence Interval
DDS	Delirium Detection Score
DOSS	Delirium Observation Screening Scale
DSM-5	Diagnostic and Statistical Manual of Mental Disorders, Fifth Edition
EG	Experimental Group
ERAS	Enhanced Recovery After Surgery
HELP	Hospital Elder Life Program
ICDSC	Intensive Care Delirium Screening Checklist
ICU	Intensive Care Unit
MD	Mean Difference
Nu-DESC	Nursing Delirium Screening Scale
OPCABG	Off-Pump Coronary Artery Bypass Grafting
PICOS	Population, Intervention, Comparator, Outcomes, and Study Design
PRISMA	Preferred Reporting Items for Systematic Reviews and Meta-Analyses
PROSPERO	International Prospective Register of Systematic Reviews
RASS	Richmond Agitation-Sedation Scale
RCT	Randomized Controlled Trial
RoB 2	Risk of Bias 2
ROBVIS	Risk-of-Bias Visualization
RR	Risk Ratio
SCCM	Society of Critical Care Medicine
SMD	Standardized Mean Difference
TAES	Transcutaneous Acupoint Electrical Stimulation
4AT	4 A’s Test

## References

[1] Yan E., Veitch M., Saripella A., Alhamdah Y., Butris N., Tang-Wai D.F., Tartaglia M.C., Nagappa M., Englesakis M., He D. (2023). Association between Postoperative Delirium and Adverse Outcomes in Older Surgical Patients: A Systematic Review and Meta-Analysis. J. Clin. Anesth..

[2] Jin Z., Hu J., Ma D. (2020). Postoperative Delirium: Perioperative Assessment, Risk Reduction, and Management. Br. J. Anaesth..

[3] Mevorach L., Forookhi A., Farcomeni A., Romagnoli S., Bilotta F. (2023). Perioperative Risk Factors Associated with Increased Incidence of Postoperative Delirium: Systematic Review, Meta-Analysis, and Grading of Recommendations Assessment, Development, and Evaluation System Report of Clinical Literature. Br. J. Anaesth..

[4] Devlin J.W., Skrobik Y., Gélinas C., Needham D.M., Slooter A.J.C., Pandharipande P.P., Watson P.L., Weinhouse G.L., Nunnally M.E., Rochwerg B. (2018). Clinical Practice Guidelines for the Prevention and Management of Pain, Agitation/Sedation, Delirium, Immobility, and Sleep Disruption in Adult Patients in the ICU. Crit. Care Med..

[5] Barr J., Downs B., Ferrell K., Talebian M., Robinson S., Kolodisner L., Kendall H.R., Holdych J.P. (2024). Improving Outcomes in Mechanically Ventilated Adult ICU Patients Following Implementation of the ICU Liberation (ABCDEF) Bundle across a Large Healthcare System. Crit. Care Explor..

[6] Johnson G.U., Towell-Barnard A., McLean C., Ewens B. (2024). Delirium Prevention and Management in an Adult Intensive Care Unit through Evidence-Based Nonpharmacological Interventions: A Scoping Review. Collegian.

[7] Wang J., Lu Y., Chen X., Wu Y. (2024). Effectiveness of Nurse-Led Non-Pharmacological Interventions on Outcomes of Delirium in Adults: A Meta-Analysis of Randomized Controlled Trials. Worldviews Evid. Based Nurs..

[8] Lange S., Mędrzycka-Dąbrowska W., Friganović A., Oomen B., Krupa S. (2022). Non-Pharmacological Nursing Interventions to Prevent Delirium in ICU Patients—An Umbrella Review with Implications for Evidence-Based Practice. J. Pers. Med..

[9] Li J., Fan Y., Luo R., Yin N., Wang Y., Jing J., Zhang J. (2025). The Impact of Non-Pharmacological Sleep Interventions on Delirium Prevention and Sleep Improvement in Postoperative ICU Patients: A Systematic Review and Network Meta-Analysis. Intensive Crit. Care Nurs..

[10] Fan Y.Y., Luo R.Y., Wang M.T., Yuan C.Y., Sun Y.Y., Jing J.Y. (2024). Mechanisms Underlying Delirium in Patients with Critical Illness. Front. Aging Neurosci..

[11] Ali M., Cascella M. (2025). ICU Delirium. StatPearls.

[12] Golubovic J., Neerland B., Aune D., Baker F.A. (2022). Music Interventions and Delirium in Adults: A Systematic Review and Meta-Analysis. Brain Sci..

[13] Page M.J., McKenzie J.E., Bossuyt P.M., Boutron I., Hoffmann T.C., Mulrow C.D., Shamseer L., Tetzlaff J.M., Akl E.A., Brennan S.E. (2021). The PRISMA 2020 Statement: An Updated Guideline for Reporting Systematic Reviews. PLoS Med..

[14] Barr J., Fraser G.L., Puntillo K., Ely E.W., Gélinas C., Dasta J.F., Davidson J.E., Devlin J.W., Kress J.P., Joffe A.M. (2013). Clinical Practice Guidelines for the Management of Pain, Agitation, and Delirium in Adult Patients in the Intensive Care Unit. Crit. Care Med..

[15] Ouzzani M., Hammady H., Fedorowicz Z., Elmagarmid A. (2016). Rayyan—A Web and Mobile App for Systematic Reviews. Syst. Rev..

[16] Higgins J.P.T., Thomas J., Chandler J., Cumpston M., Li T., Page M.J., Welch V.A. (2019). Cochrane Handbook for Systematic Reviews of Interventions.

[17] Sterne J.A.C., Savović J., Page M.J., Elbers R.G., Blencowe N.S., Boutron I., Cates C.J., Cheng H.Y., Corbett M.S., Eldridge S.M. (2019). RoB 2: A Revised Tool for Assessing Risk of Bias in Randomised Trials. Br. Med. J..

[18] Sterne J.A.C., Hernán M.A., Reeves B.C., Savović J., Berkman N.D., Viswanathan M., Henry D., Altman D.G., Ansari M.T., Boutron I. (2016). ROBINS-I: A Tool for Assessing Risk of Bias in Non-Randomised Studies of Interventions. Br. Med. J..

[19] Guo Y., Fan Y. (2016). A preoperative, nurse-led intervention program reduces acute postoperative delirium. J. Neurosci. Nurs..

[20] Patel J., Baldwin J., Bunting P., Laha S. (2014). The effect of a multicomponent multidisciplinary bundle of interventions on sleep and delirium in medical and surgical intensive care patients. Anaesthesia.

[21] Welsch E., Vashisht A., Stutzman S.E., Olson D.M. (2023). Family presence may reduce postoperative delirium after spinal surgery. J. Neurosci. Nurs..

[22] Zhang W., Sun Y., Liu Y., Qiu W., Ye X., Zhang G., Zhang L. (2017). A nursing protocol targeting risk factors for reducing postoperative delirium in patients following coronary artery bypass grafting: Results of a prospective before-after study. Int. J. Nurs. Sci..

[23] Chen L., Zheng J., Lv S., Li B., Yang L. (2021). Impact of a sleep promotion protocol on off-pump coronary artery bypass graft patients. Nurs. Crit. Care.

[24] McGuinness L.A., Higgins J.P.T. (2021). Risk-of-Bias VISualization (robvis): An R Package and Shiny Web App for Visualizing Risk-of-Bias Assessments. Res. Synth. Methods.

[25] R Core Team (2024). R: A Language and Environment for Statistical Computing.

[26] Balduzzi S., Rücker G., Schwarzer G. (2019). How to Perform a Meta-Analysis with R: A Practical Tutorial. Evid. Based Ment. Health.

[27] Röver C., Knapp G., Friede T. (2015). Hartung-Knapp-Sidik-Jonkman Approach and Its Modification for Random-Effects Meta-Analysis with Few Studies. BMC Med. Res. Methodol..

[28] Wan X., Wang W., Liu J., Tong T. (2014). Estimating the Sample Mean and Standard Deviation from the Sample Size, Median, Range and/or Interquartile Range. BMC Med. Res. Methodol..

[29] Duval S., Tweedie R. (2000). Trim and Fill: A Simple Funnel-Plot-Based Method of Testing and Adjusting for Publication Bias in Meta-Analysis. Biometrics.

[30] Hu R.F., Jiang X.Y., Hegadoren K.M., Zhang Y.H. (2015). Effects of earplugs and eye masks combined with relaxing music on sleep, melatonin and cortisol levels in ICU patients: A randomized controlled trial. Crit. Care.

[31] Kim J., Choi D., Yeo M.S., Yoo G.E., Kim S.J., Na S. (2020). Effects of patient-directed interactive music therapy on sleep quality in postoperative elderly patients: A randomized-controlled trial. Nat. Sci. Sleep.

[32] Erbay Dallı Ö., Yıldırım Y., Şenuzun Aykar F., Kahveci F. (2023). The effect of music on delirium, pain, sedation and anxiety in patients receiving mechanical ventilation in the intensive care unit. Intensive Crit. Care Nurs..

[33] Topal T., Sürme Y. (2025). The effect of music and eye masks on sleep quality and delirium in abdominal surgery intensive care patients: Randomized controlled trial. Nurs. Crit. Care.

[34] Jiang Y., Xie Y., Fang P., Shang Z., Chen L., Zhou J., Yang C., Zhu W., Hao X., Ding J. (2024). Cognitive training for reduction of delirium in patients undergoing cardiac surgery: A randomized clinical trial. JAMA Netw. Open.

[35] Kappen P.R., Mos M.I., Jeekel J., Dirven C.M.F., Kushner S.A., Osse R.J., Coesmans M., Poley M.J., van Schie M.S., van der Holt B. (2023). Music to prevent deliriUm during neuroSurgerY (MUSYC): A single-centre, prospective randomised controlled trial. BMJ Open.

[36] Cao C., Gao D., Wang X., Wan Y., Zhu Y., Yao S., Wang R. (2023). Analysis of the influencing factors of delirium after PCI for acute myocardial infarction and its impact on the quality of life of patients. Altern. Ther. Health Med..

[37] Méndez-Martínez C., Casado-Verdejo I., Fernández-Fernández J.A., Sánchez-Valdeón L., Bello-Corral L., Méndez-Martínez S., Sandoval-Diez A., Gómez-Salgado J., García-Suárez M., Fernández-García D. (2024). Projection of visual material on postoperative delirium in patients undergoing cardiac surgery: A double blind randomized clinical trial. Medicine.

[38] Xu J., Yu W., Yu Y., Zhou J., Deng M., Wang W., Jiang J., Chang W., Yang Y., Cao H. (2026). The effect of early mobilization of post-cardiac surgery patients on delirium: A pilot study. Eur. J. Phys. Rehabil. Med..

[39] Moon K.J., Lee S.M. (2015). The effects of a tailored intensive care unit delirium prevention protocol: A randomized controlled trial. Int. J. Nurs. Stud..

[40] Gómez Tovar L.O., Henao Castaño A.M. (2024). Dynamic delirium—Nursing intervention to reduce delirium in patients critically ill, a randomized control trial. Intensive Crit. Care Nurs..

[41] Khan S.H., Xu C., Purpura R., Durrani S., Lindroth H., Wang S., Gao S., Heiderscheit A., Chlan L., Boustani M. (2020). Decreasing delirium through music: A randomized pilot trial. Am. J. Crit. Care.

[42] Abbasinia M., Babaii A., Nadali Z., Pakzaban S., Abbasi M., Shamali M. (2021). The effects of a tailored postoperative delirium prevention intervention after coronary artery bypass graft: A randomised controlled trial. Nurs. Pract. Today.

[43] Giraud K., Pontin M., Sharples L.D., Fletcher P., Dalgleish T., Eden A., Jenkins D.P., Vuylsteke A. (2016). Use of a structured mirrors intervention does not reduce delirium incidence but may improve factual memory encoding in cardiac surgical ICU patients aged over 70 years: A pilot time-cluster randomized controlled trial. Front. Aging Neurosci..

[44] Lei L., Katznelson R., Fedorko L., Carroll J., Poonawala H., Machina M., Styra R., Rao V., Djaiani G. (2017). Cerebral oximetry and postoperative delirium after cardiac surgery: A randomised, controlled trial. Anaesthesia.

[45] Xue X., Wang P., Wang J., Li X., Peng F., Wang Z. (2020). Preoperative individualized education intervention reduces delirium after cardiac surgery: A randomized controlled study. J. Thorac. Dis..

[46] Kasapoğlu E.S., Enç N. (2022). Role of multicomponent non-pharmacological nursing interventions on delirium prevention: A randomized controlled study. Geriatr. Nurs..

[47] Esfahanian F., Mirmohammadsadeghi A., Gholami H., Neshat S., Mansouri M., Sadeghi M., Bathaie S.R., Heidari Z., Mirmohammadsadeghi M. (2022). Using music for the prevention of delirium in patients after coronary artery bypass graft surgery: A randomized clinical trial. J. Cardiothorac. Vasc. Anesth..

[48] Kitisin N., Lao-Amornphunkul S., Hemtanon N., Thikom N., Phochan N., Thanakiattiwibun C., Chaiwat O., Wongtangman K., Trachuthamcharoen P., Piriyapatsom A. (2025). Effects of a multicomponent sleep enhancement protocol on delirium incidence in elderly critically ill surgical patients: A randomized controlled trial. J. Intensive Care.

[49] Zhang Y., Gong L., Zhang Y., Dong S., Shi J., Li X., Guo Y., He S., Wu X., Liu S. (2022). Effect of transcutaneous acupoint electrical stimulation on urinary retention and urinary ATP in elderly patients after laparoscopic cholecystectomy: A prospective, randomized, controlled clinical trial. Clin. Interv. Aging.

[50] Faustino T.N., Suzart N.A., Rabelo R.N.S., Santos J.L., Batista G.S., Freitas Y.S., Saback D.A., Sales N.M.M.D., Barreto B.B., Gusmao-Flores D. (2022). Effectiveness of combined non-pharmacological interventions in the prevention of delirium in critically ill patients: A randomized clinical trial. J. Crit. Care.

