# Supplementary material for: Effectiveness of Non-Pharmacological Interventions for the Prevention of Postoperative Delirium in Critically Ill Adult Patients: A Systematic Review and Meta-Analysis
Source: Nurs Rep. 2026 Sep 8;16(9):328. doi: 10.3390/nursrep16090328 (PMC13610546; doi:10.3390/nursrep16090328)
Supplement: Supplementary file 1 [file nursrep-16-00328-s001.zip › nursrep-4457034-supplementary.pdf]

Supplementary Materials

Table S1. Search strategies by database

Search update: All information sources were last searched on 29 May 2026.

Database / Information source	Platform / Interface	Search strategy	Limits	Last searched
PubMed/MEDLINE	PubMed	("Delirium"[MeSH] OR delirium[tiab] OR "acute confusion"[tiab] OR "acute confus*"[tiab]) AND (postoperative[tiab] OR postoperativ*[tiab] OR "Postoperative Period"[MeSH] OR surgery[tiab] OR surgical[tiab]) AND ("Intensive Care Units"[MeSH] OR ICU[tiab] OR "critical care"[tiab] OR "critical care unit"[tiab]) AND (nonpharmacologic*[tiab] OR "non-pharmacologic*"[tiab] OR bundle*[tiab] OR ABCDEF[tiab] OR "ICU Liberation"[tiab] OR mobilization[tiab] OR mobilisation[tiab] OR "early mobil*"[tiab] OR "sleep hygiene"[tiab] OR circadian[tiab] OR light[tiab] OR noise[tiab] OR reorientation[tiab] OR reorient*[tiab] OR "cognitive stimulation"[tiab] OR sensory[tiab] OR "hearing aid*"[tiab] OR eyeglass*[tiab] OR "family engagement"[tiab] OR family[tiab] OR music*[tiab] OR "music therapy"[tiab] OR "sedation protocol"[tiab] OR "light sedation"[tiab])	Publication date: 2014/01/01 to 2026/05/29; English or Spanish	29 May 2026
Embase	Ovid	('delirium'/exp OR delirium:ti,ab OR 'acute confusion':ti,ab) AND ('postoperative period'/exp OR postoperative:ti,ab OR postoperativ*:ti,ab OR surgery:ti,ab OR surgical:ti,ab) AND ('intensive care unit'/exp OR icu:ti,ab OR 'critical care':ti,ab OR 'critical care unit':ti,ab) AND (nonpharmacologic*:ti,ab OR 'non-pharmacologic*':ti,ab OR bundle*:ti,ab OR abcdef:ti,ab OR	Publication years: 2014–2026; English or Spanish; adult population	29 May 2026

		'icu liberation':ti,ab OR mobilization:ti,ab OR mobilisation:ti,ab OR 'early mobilization':ti,ab OR 'sleep hygiene':ti,ab OR circadian:ti,ab OR light:ti,ab OR noise:ti,ab OR reorientation:ti,ab OR 'cognitive stimulation':ti,ab OR sensory:ti,ab OR 'hearing aid*':ti,ab OR eyeglass*:ti,ab OR 'family engagement':ti,ab OR music*:ti,ab OR 'music therapy'/exp OR 'sedation protocol':ti,ab OR 'light sedation':ti,ab)		
CINAHL	EBSCOhost	(MH "Delirium" OR TI delirium OR AB delirium OR AB "acute confusion") AND (MH "Postoperative Period" OR TI postoperative OR AB postoperative OR TI surgery OR AB surgery) AND (MH "Intensive Care Units" OR TI ICU OR AB ICU OR TI "critical care" OR AB "critical care") AND (TI nonpharmacologic* OR AB nonpharmacologic* OR TI bundle* OR AB bundle* OR AB ABCDEF OR AB "ICU Liberation" OR TI mobilization OR AB mobilization OR TI mobilisation OR AB mobilisation OR TI "sleep hygiene" OR AB "sleep hygiene" OR AB circadian OR TI light OR AB light OR TI noise OR AB noise OR TI reorientation OR AB reorientation OR AB "cognitive stimulation" OR TI sensory OR AB sensory OR AB "family engagement" OR TI music OR AB music OR AB "music therapy" OR TI "sedation protocol" OR AB "light sedation")	Published date: 2014/01/01 to 2026/05/29; English or Spanish; peer-reviewed	29 May 2026
Cochrane CENTRAL	Cochrane Library	(delirium OR "acute confusion" OR "postoperative delirium") AND (postoperative OR surgery OR "postoperative period") AND ("intensive care" OR ICU OR "critical care" OR "critical care unit") AND (nonpharmacologic OR "non pharmacologic" OR "non-pharmacologic" OR bundle OR ABCDEF OR "ICU Liberation" OR mobilisation OR mobilization OR "sleep hygiene" OR circadian OR reorientation OR "cognitive	Publication years: 2014–2026; adults	29 May 2026

		stimulation" OR sensory OR "family engagement" OR music OR "music therapy" OR "sedation protocol" OR "light sedation")		
Web of Science	Web of Science Core Collection	TS=(delirium OR "acute confusion" OR "postoperative delirium") AND TS=(postoperative OR postoperativ* OR "postoperative period" OR surgery OR surgical) AND TS=("intensive care" OR ICU OR "critical care" OR "critical care unit") AND TS=(nonpharmacologic OR "non pharmacologic" OR "non-pharmacologic" OR bundle OR ABCDEF OR "ICU Liberation" OR mobilisation OR mobilization OR "sleep hygiene" OR circadian OR reorientation OR "cognitive stimulation" OR sensory OR "family engagement" OR music OR "music therapy" OR "sedation protocol" OR "light sedation")	Publication years: 2014–2026; English or Spanish	29 May 2026
Scopus	Scopus	TITLE-ABS-KEY(delirium OR "acute confusion" OR "postoperative delirium") AND TITLE-ABS-KEY(postoperative OR postoperativ* OR "postoperative period" OR surgery OR surgical) AND TITLE-ABS-KEY("intensive care" OR ICU OR "critical care" OR "critical care unit") AND TITLE-ABS-KEY(nonpharmacologic OR "non pharmacologic" OR "non-pharmacologic" OR bundle OR ABCDEF OR "ICU Liberation" OR mobilization OR mobilisation OR "sleep hygiene" OR circadian OR reorientation OR "cognitive stimulation" OR sensory OR "family engagement" OR music OR "music therapy" OR "sedation protocol" OR "light sedation")	PUBYEAR > 2013 AND PUBYEAR < 2027; English or Spanish	29 May 2026
LILACS	Virtual Health Library (VHL)	(delirio OR delirium OR "confusión aguda") AND (postoperatorio OR postoperatoria OR "periodo postoperatorio" OR "postoperative period" OR cirugía) AND ("unidad de cuidados intensivos" OR UCI OR "cuidados críticos" OR "intensive care" OR ICU) AND ("intervención no farmacológica" OR "no farmacológico" OR bundle OR "movilización precoz" OR "higiene	Publication years: 2014–2026; Spanish or English	29 May 2026

		del sueño" OR "control del ruido" OR "reorientación cognitiva" OR "participación familiar" OR "terapia musical" OR "sedación ligera")		
Google Scholar	Google Scholar	"postoperative delirium" AND ("intensive care" OR ICU OR "critical care") AND ("non-pharmacological intervention" OR "nonpharmacological intervention" OR bundle OR mobilization OR mobilisation OR "sleep hygiene" OR "family engagement" OR "music therapy" OR "light sedation" OR "ICU Liberation")	Publication years: 2014–2026. Results were examined according to the relevance ranking provided by Google Scholar. The first 320 records returned by the search were retrieved and incorporated into the deduplication and screening workflow. No additional eligibility restrictions specific to Google Scholar were applied.	29 May 2026

Table S2. Meta-analyses of secondary outcomes associated with non-pharmacological interventions for postoperative delirium prevention

Study	Year	Experimental	Control	Place	n exp	n control	Estimator	lower limit	upper limit	Weight (random)
**Severity (I^2 = 82.7%; τ^2 = 0.45; p = 0.323)**					**127**	**114**	**-0.56**	**-2.40**	**1.29**	
Topal 2025	2025	Sleep / music / sensory optimization	Routine care	Kayseri, Türkiye	21	24	-1.43	-2.09	-0.77	30.9
Khan 2020	2020	Music Therapy	Audiobook	United States	34	18	0.04	-0.54	0.61	32.6

Faustino 2022	2022	Multi-component bundle	Routine care	Salvador, Brazil	72	72	-0.35	-0.68	-0.02	36.5
**Duration (I² = 83.5%; τ² = 0.68; p = 0.149)**					**289**	**286**	**-1.17**	**-3.38**	**1.03**	
Jiang 2024	2024	Cognitive training	Routine care	Southeastern China	102	106	-0.34	-0.66	-0.02	41.2
Giraud 2016	2016	Sensory optimization	Routine care	Cambridge, United Kingdom	115	108	-2	-3.02	-0.98	30.6
Faustino 2022	2022	Multi-component bundle	Routine care	Salvador, Brazil	72	72	-1.5	-2.66	-0.34	28.2
**Days in the intensive care unit (I² = 24.4%; τ² = 0.06; p = 0.320)**					**277**	**273**	**-0.18**	**-0.65**	**0.30**	
Moon 2015	2015	Individualized delirium prevention protocol	Routine care	Seoul, South Korea	60	63	0.8	-3.01	4.61	1.0
Abbasinia 2021	2021	Educational Video + Hospital Elder Life Program	Routine care	Qom, Iran	30	30	-0.53	-1.03	-0.03	31.1
Giraud 2016	2016	Sensory optimization	Routine care	Cambridge, United Kingdom	115	108	0	-0.19	0.19	56.4
Faustino 2022	2022	Multi-component bundle	Routine care	Salvador, Brazil	72	72	-0.17	-1.2	0.86	11.5

For the severity outcome, the standardized mean difference (SMD) was used because the original studies employed different scales. For the duration and ICU days outcomes, the mean difference was used, as the same scales were applied in each case.
lower limit: lower limit of the 95% confidence interval; upper limit: upper limit of the 95% confidence interval.
REML and Hartung–Knapp test.

Table S3. PRISMA 2020 checklist indicating where each reporting item is addressed in the manuscript

Line-number reference: line numbers correspond to the final line-numbered manuscript accompanying this supplementary file. **Status:** X = reported; N/A = not applicable because the analysis was not conducted; Partial = incompletely reported in the available manuscript materials.

Section and topic	Item	Checklist item	Status	Location where item is reported
TITLE	1	Identify the report as a systematic review.	X	Title, lines 3–5.
ABSTRACT	2	See the PRISMA 2020 for Abstract's checklist.	X	Abstract, lines 15–45.
INTRODUCTION — Rationale	3	Describe the rationale for the review in the context of existing knowledge.	X	Introduction, lines 49–116.
INTRODUCTION — Objectives	4	Provide an explicit statement of the objective(s) or question(s) the review addresses.	X	Introduction, lines 117–119.
METHODS — Eligibility criteria	5	Specify the inclusion and exclusion criteria for the review and how studies were grouped for the syntheses.	X	Sections 2.2–2.3, lines 138–188.
METHODS — Information sources	6	Specify all databases, registers, websites, organisations, reference lists and other sources searched or consulted to identify studies. Specify the date when each source was last searched or consulted.	X	Section 2.4, lines 190–193; Supplementary Table S1.
METHODS — Search strategy	7	Present the full search strategies for all databases, registers and websites, including any filters and limits used.	X	Section 2.4, lines 199–213; Supplementary Table S1.
METHODS — Selection process	8	Specify the methods used to decide whether a study met the inclusion criteria, including how many reviewers screened each record and each report retrieved, whether they worked independently, and, if applicable, details of automation tools used in the process.	X	Section 2.5, Figure 1; lines 215–224.
METHODS — Data collection process	9	Specify the methods used to collect data from reports, including how many reviewers collected data from each report, whether they worked independently, any processes for obtaining or confirming data from study investigators, and, if applicable, details of automation tools used in the process.	X	Section 2.6, lines 226–234.

Section and topic	Item	Checklist item	Status	Location where item is reported
METHODS — Data items	10a	List and define all outcomes for which data were sought. Specify whether all results that were compatible with each outcome domain in each study were sought, and, if not, the methods used to decide which results to collect.	X	Section 2.2, lines 138–166
METHODS — Data items	10b	List and define all other variables for which data were sought. Describe any assumptions made about missing or unclear information.	X	Section 2.6, lines 228–234.
METHODS — Study risk of bias assessment	11	Specify the methods used to assess risk of bias in the included studies, including details of the tool(s) used, how many reviewers assessed each study and whether they worked independently, and, if applicable, details of automation tools used in the process.	X	Section 2.7, lines 236–253.
METHODS — Effect measures	12	Specify for each outcome the effect measure(s) used in the synthesis or presentation of results.	X	Section 2.8, lines 256–305.
METHODS — Synthesis methods	13a	Describe the processes used to decide which studies were eligible for each synthesis.	X	Section 2.8, lines 256–305.
METHODS — Synthesis methods	13b	Describe any methods required to prepare the data for presentation or synthesis, such as handling of missing summary statistics or data conversions.	X	Section 2.8, lines 256–305.
METHODS — Synthesis methods	13c	Describe any methods used to tabulate or visually display results of individual studies and syntheses.	X	Table 1; Table 2; Figures 2–4; Supplementary Table S2.
METHODS — Synthesis methods	13d	Describe any methods used to synthesize results and provide a rationale for the choice(s). If meta-analysis was performed, describe the model(s), method(s) used to identify statistical heterogeneity, and software package(s) used.	X	Section 2.8, lines 256–305. New statistical methods.
METHODS — Synthesis methods	13e	Describe any methods used to explore possible causes of heterogeneity among study results.	X	Section 2.8, lines 256–305. Subgroup analysis.

Section and topic	Item	Checklist item	Status	Location where item is reported
METHODS — Synthesis methods	13f	Describe any sensitivity analyses conducted to assess robustness of the synthesized results.	X	Section 2.8; Section 3.7; Supplementary Table S5, Panels A–C.
METHODS — Reporting bias assessment	14	Describe any methods used to assess risk of bias due to missing results in a synthesis.	X	Section 2.8, Statistical Analysis; Section 3.6, Publication Bias; Figure 4.
METHODS — Certainty assessment	15	Describe any methods used to assess certainty (or confidence) in the body of evidence for an outcome.	X	Section 2.8; lines 241-242.
RESULTS — Study selection	16a	Describe the results of the search and selection process, from the number of records identified in the search to the number of studies included in the review, ideally using a flow diagram.	X	Section 3.1, line 332; Figure 1.
RESULTS — Study selection	16b	Cite studies that might appear to meet the inclusion criteria, but which were excluded, and explain why they were excluded.	X	Results, Section 3.1, Study Selection; Supplementary Table S4.
RESULTS — Study characteristics	17	Cite each included study and present its characteristics.	X	Section 3.2, lines 275–307; Table 1.
RESULTS — Risk of bias in studies	18	Present assessments of risk of bias for each included study.	X	Section 3.3, lines 316–342; Figure 2.
RESULTS — Results of individual studies	19	For all outcomes, present, for each study, summary statistics for each group and an effect estimate and its precision, ideally using structured tables or plots.	X	Section 3.4, lines 341–390; Table 2; Figure 3; Supplementary Table S2; Table 2 and Figure 3 for the primary outcome; Supplementary Table S2 for the meta-analysed secondary outcomes.
RESULTS — Results of syntheses	20a	For each synthesis, briefly summarise the characteristics and risk of bias among contributing studies.	X	Sections 3.2–3.4, line 390.
RESULTS — Results of syntheses	20b	Present results of all statistical syntheses conducted. If meta-analysis was done, present the summary estimate and its precision and measures of statistical heterogeneity.	X	Sections 3.5 and 3.7, line 473; Figure 3; Supplementary Table S2.
RESULTS — Results of syntheses	20c	Present results of all investigations of possible causes of heterogeneity among study results.	X	Section 3.5 Subgroup results, line 390

Section and topic	Item	Checklist item	Status	Location where item is reported
RESULTS — Results of syntheses	20d	Present results of all sensitivity analyses conducted to assess the robustness of the synthesized results.	X	Section 3.7; Supplementary Table S5, Panels A–C.
RESULTS — Reporting biases	21	Present assessments of risk of bias due to missing results for each synthesis assessed.	X	Section 3.6, Publication Bias Assessment; Figure 4
RESULTS — Certainty of evidence	22	Present assessments of certainty (or confidence) in the body of evidence for each outcome assessed.	X	Section 3.8, lines 403-420; Table 3; Supplementary Table S6. Updated GRADE line 584
DISCUSSION — Discussion	23a	Provide a general interpretation of the results in the context of other evidence.	X	Sections 4.1–4.3, lines 600–615.
DISCUSSION — Discussion	23b	Discuss any limitations of the evidence included in the review.	X	Section 4.5, lines 621–654.
DISCUSSION — Discussion	23c	Discuss any limitations of the review processes used.	X	Section 4.5, lines 802-871.
DISCUSSION — Discussion	23d	Discuss implications of the results for practice, policy, and future research.	X	Section 4.4, lines 739–800; Conclusions, lines 872–888.
OTHER INFORMATION — Registration and protocol	24a	Provide registration information for the review, including register name and registration number, or state that the review was not registered.	X	Section 2.1, Design and Registration; PROSPERO CRD420251247117.
OTHER INFORMATION — Registration and protocol	24b	Indicate where the review protocol can be accessed, or state that a protocol was not prepared.	X	Section 2.1, Design and Registration; PROSPERO CRD420251247117.
OTHER INFORMATION — Registration and protocol	24c	Describe and explain any amendments to information provided at registration or in the protocol.	X	Section 2.1 and Supplementary Table S7. The PROSPERO registration contained internally inconsistent entries regarding age, eligible study designs, and search date restrictions. The final operational criteria and deviations are reported explicitly. Prespecified and post hoc analyses are distinguished in Section 2.8 and Supplementary Table S7.
OTHER INFORMATION — Support	25	Describe sources of financial or non-financial support for the review, and the role of the funders or sponsors.	X	Funding statement, line 906.

Section and topic	Item	Checklist item	Status	Location where item is reported
OTHER INFORMATION — Competing interests	26	Declare any competing interests of review authors.	X	Conflicts of Interest, line 923.
OTHER INFORMATION — Availability of data, code and other materials	27	Report which materials are publicly available and where they can be found, including data collection forms, extracted data, analysis data, analytic code, and other materials used in the review.	X	Data Availability Statement; extracted meta-analysis data are available from the corresponding author upon reasonable request.

Attribution: Adapted from the PRISMA 2020 checklist: Page, M.J.; McKenzie, J.E.; Bossuyt, P.M.; Boutron, I.; Hoffmann, T.C.; Mulrow, C.D.; Shamseer, L.; Tetzlaff, J.M.; Akl, E.A.; Brennan, S.E.; et al. The PRISMA 2020 Statement: An Updated Guideline for Reporting Systematic Reviews. PLoS Med. 2021, 18, e1003583. https://doi.org/10.1371/journal.pmed.1003583**.** The checklist is distributed under the Creative Commons Attribution 4.0 International License.

Table S4. Reports excluded after full-text assessment and primary reasons for exclusion

Each report was assigned one mutually exclusive primary reason for exclusion. The category totals correspond to the PRISMA 2020 flow diagram (n = 82).

No.	Excluded report	Primary reason for exclusion
1	Mills GH et al. Do earplugs stop noise from driving critical care patients into delirium? CRITICAL CARE. 2012;16(4). doi:10.1186/cc11397.	Wrong study design
2	Ettema RGA et al. Development of a nursing intervention to prepare frail older patients for cardiac surgery (the PREDOCS programme), following phase one of the guidelines of the Medical Research Council. EUROPEAN JOURNAL OF CARDIOVASCULAR NURSING. 2014;13(6):494-505. doi:10.1177/1474515113511715.	Wrong study design
3	Simons KS et al. Dynamic light application therapy to reduce the incidence and duration of delirium in intensive-care patients: a randomised controlled trial. The Lancet Respiratory Medicine. 2016;4(3):194-202. doi:10.1016/S2213-2600(16)00025-4.	Wrong population
4	Mailhot T et al. A post cardiac surgery intervention to manage delirium involving families: a randomized pilot study. NURSING IN CRITICAL CARE. 2017;22(4):221-228. doi:10.1111/nicc.12288.	Wrong study design
5	Mitchell ML et al. A family intervention to reduce delirium in hospitalised ICU patients: A feasibility randomised controlled trial. Intensive and Critical Care Nursing. 2017; 40:77-84. doi: 10.1016/j.iccn.2017.01.001.	Wrong population
6	Menger J et al. Earplugs during the first night after cardiothoracic surgery may improve a fast-track protocol. MINERVA ANESTESIOLOGICA. 2018;84(1):49-57. doi:10.23736/S0375-9393.17.11758-X.	Wrong study design
7	Fahimi K et al. The effects of multimedia education on postoperative delirium in patients undergoing coronary artery bypass graft: A randomized clinical trial. NURSING IN CRITICAL CARE. 2020;25(6):346-352. doi:10.1111/nicc.12473.	Wrong study design
8	Ortiz D. Assessment and management of agitation, sleep, and mental illness in the surgical ICU. CURRENT OPINION IN CRITICAL CARE. 2020;26(6):634-639. doi:10.1097/MCC.0000000000000762.	Wrong study design
9	Kim H et al. Effect on Quality of Care of a Delirium Prevention Campaign for Surgical Intensive Care Nurses. JOURNAL OF NURSING CARE QUALITY. 2021;36(4):361-368. doi:10.1097/NCQ.0000000000000535.	Wrong study design
10	Rood PJT et al. The Impact of Nursing Delirium Preventive Interventions in the ICU A Multicenter Cluster-randomized Controlled Clinical Trial. AMERICAN JOURNAL OF RESPIRATORY AND CRITICAL CARE MEDICINE. 2021;204(6):682-691. doi:10.1164/rccm.202101-0082OC.	Wrong population
11	Unal N et al. Evaluation of the effectiveness of delirium prevention care protocol for the patients with hip fracture: A randomised controlled study. JOURNAL OF CLINICAL NURSING. 2022;31(7):1082-1094. doi:10.1111/jocn.15973.	Wrong setting
12	Deemer K et al. Occupational therapist-guided cognitive interventions in critically ill patients: a feasibility randomized controlled trial. Canadian journal of anaesthesia = Journal canadien d'anesthesie. 2023;70(1):139-150. doi:10.1007/s12630-022-02351-9.	Wrong population

13	Gomez Tovar LO et al. Effectiveness of nursing intervention to reduce delirium in adult critically ill- A protocol for a randomized trial. CONTEMPORARY CLINICAL TRIALS COMMUNICATIONS. 2023;31. doi:10.1016/j.conctc.2022.101042.	Wrong study design
14	Liang S et al. The effects of a sensory stimulation intervention for preventing delirium in a surgical intensive care unit: A randomized controlled trial. NURSING IN CRITICAL CARE. 2023;28(5):709-717. doi:10.1111/nicc.12913.	Wrong study design
15	Hashemi S et al. The Effect of Multidimensional Nursing Interventions on the Incidence of Delirium in Patients with COVID-19. IRANIAN JOURNAL OF NURSING AND MIDWIFERY RESEARCH. 2024;29(6):714-719. doi:10.4103/ijnmr.ijnmr_59_23.	Wrong study design
16	Shorofi SA et al. The efficacy of earplugs and eye masks for delirium severity and sleep quality in patients undergoing coronary artery bypass grafting in cardiac intensive care units: A single-blind, randomised controlled trial. Australian Critical Care. 2024;37(1):74-83. doi:10.1016/j.aucc.2023.08.003.	Wrong study design
17	Burzlaff P et al. Vibroacoustic music therapy for postoperative delirium in the intensive care unit (VIMUD-ICU): Study protocol for a pragmatic randomized controlled trial. NORDIC JOURNAL OF MUSIC THERAPY. 2025. doi:10.1080/08098131.2025.2578850.	Wrong study design
18	Fan Q et al. Association Between Plasma Amyloid-Beta 42 Ratio and Postoperative Delirium in Elderly Patients Undergoing Major Abdominal Surgery: Secondary Analysis of a Randomized Controlled Trial. Brain and behavior. 2025;15(4):e70501. doi:10.1002/brb3.70501.	Wrong study design
19	Ozdemir FE et al. Effects of Psychosocial Nursing Interventions on Sleep, Anxiety and Delirium in Patients Undergoing Open Heart Surgery: A Randomised Controlled Trial. NURSING IN CRITICAL CARE. 2025;30(6). doi:10.1111/nicc.70216.	Wrong study design
20	Bennion J et al. Does in-bed cycling delivered within 48 hours of mechanical ventilation, reduce the occurrence of delirium in critically ill patients: A mixed-methods feasibility randomised controlled trial protocol. Journal of the Intensive Care Society. 2026;27(1):90-97. doi:10.1177/17511437251400612.	Wrong study design
21	Tan Q et al. Effect of intraoperative 40-hz gamma-frequency auditory stimulation on postoperative delirium in older adults undergoing major surgery: a randomized clinical trial protocol. Annals of medicine. 2026;58(1):2657098. doi:10.1080/07853890.2026.2657098.	Wrong study design
22	Guided meditation as an adjunct to enhance postoperative recovery after cardiac surgery: study protocol for a prospective randomized controlled feasibility trial. doi:10.1186/s13063-018-3103-8.	Wrong study design
23	Bigatello LM et al. Effects of routine monitoring of delirium in a surgical/trauma intensive care unit. JOURNAL OF TRAUMA AND ACUTE CARE SURGERY. 2013;74(3):876-883. doi:10.1097/TA.0b013e31827e1b69.	Wrong outcome
24	Chevillon C et al. PREOPERATIVE EDUCATION ON POSTOPERATIVE DELIRIUM, ANXIETY, AND KNOWLEDGE IN PULMONARY THROMBO-	Wrong outcome

	ENDARTERECTOMY PATIENTS. AMERICAN JOURNAL OF CRITICAL CARE. 2015;24(2):164-171. doi:10.4037/ajcc2015658.	
25	Nickels MR et al. Critical Care Cycling Study (CYCLIST) trial protocol: a randomised controlled trial of usual care plus additional in-bed cycling sessions versus usual care in the critically ill. BMJ OPEN. 2017;7(10). doi:10.1136/bmjopen-2017-017393.	Wrong outcome
26	Rosa RG et al. Effectiveness and Safety of an Extended ICU Visitation Model for Delirium Prevention: A Before and After Study. CRITICAL CARE MEDICINE. 2017;45(10):1660-1667. doi:10.1097/CCM.0000000000002588.	Wrong outcome
27	Fraser V et al. Evaluation of an Intervention With Nurses for Delirium Detection After Cardiac Surgery. WORLDVIEWS ON EVIDENCE-BASED NURSING. 2018;15(1):38-44. doi:10.1111/wvn.12266.	Wrong outcome
28	Lisann-Goldman LR et al. Reducing Delirium and Improving Patient Satisfaction With a Perioperative Mindfulness Intervention A Mixed-Methods Pilot Study. HOLISTIC NURSING PRACTICE. 2019;33(3):163-176. doi:10.1097/HNP.0000000000000321.	Wrong outcome
29	Strike E et al. Postoperative Pain Management Strategies and Delirium After Transapical Aortic Valve Replacement: A Randomized Controlled Trial. JOURNAL OF CARDIOTHORACIC AND VASCULAR ANESTHESIA. 2019;33(6):1668-1672. doi:10.1053/j.jvca.2018.11.010.	Wrong outcome
30	Sayde GE et al. Implementing an intensive care unit (ICU) diary program at a large academic medical center: Results from a randomized control trial evaluating psychological morbidity associated with critical illness. GENERAL HOSPITAL PSYCHIATRY. 2020;66:96-102. doi:10.1016/j.genhosppsych.2020.06.017.	Wrong outcome
31	Leong RW et al. Effect of the use of earplugs and eye masks on the quality of sleep after major abdominal surgery: a randomised controlled trial. Anaesthesia. 2021;76(11):1482-1491. doi:10.1111/anae.15468.	Wrong outcome
32	Obanor OO et al. The Impact of Earplugs and Eye Masks on Sleep Quality in Surgical ICU Patients at Risk for Frequent Awakenings. CRITICAL CARE MEDICINE. 2021;49(9):E822-E832. doi:10.1097/CCM.0000000000005031.	Wrong outcome
33	Wada S et al. Yokukansan for Treatment of Preoperative Anxiety and Prevention of Postoperative Delirium in Cancer Patients Undergoing Highly Invasive Surgery. J-SUPPORT 1605 (ProD Study): A Randomized, Double-Blind, Placebo-Controlled Trial. Journal of pain and symptom management. 2021;61(1):71-80. doi:10.1016/j.jpainsymman.2020.07.009.	Wrong outcome
34	Wei X et al. Treatment of postoperative delirium with continuous theta burst stimulation: study protocol for a randomised controlled trial. BMJ open. 2021;11(8):e048093. doi:10.1136/bmjopen-2020-048093.	Wrong outcome
35	Mahesh AH et al. Impact of Physiotherapy on Functional Status and Length of Stay of Patients Admitted to Intensive Care Unit. CURRENT RESPIRATORY MEDICINE REVIEWS. 2022;18(4):267-274. doi:10.2174/1573398X18666220818095151.	Wrong outcome
36	Brennan K et al. A nurse-led intervention to reduce the incidence and duration of delirium among adults admitted to intensive care: A stepped-wedge cluster	Wrong outcome

	randomised trial. AUSTRALIAN CRITICAL CARE. 2023;36(4):441-448. doi:10.1016/j.aucc.2022.08.005.	
37	Han PH et al. Effects of an interactive handgrip game on surgical patients requiring intensive care: An assessor-blinded randomized controlled trial. INTENSIVE AND CRITICAL CARE NURSING. 2023;78. doi:10.1016/j.iccn.2023.103474.	Wrong outcome
38	Krüger L et al. Patient-related effects of primary nursing : Protocol of a pilot randomized controlled trial. Medizinische Klinik, Intensivmedizin und Notfallmedizin. 2023;118(4):257-262. doi:10.1007/s00063-023-00998-w.	Wrong outcome
39	Rapolthy-Beck A et al. Efficacy of Early Enhanced Occupational Therapy in an Intensive Care Unit (EFFORT-ICU): A Single-Site Feasibility Trial. AMERICAN JOURNAL OF OCCUPATIONAL THERAPY. 2023;77(6). doi:10.5014/ajot.2023.050230.	Wrong outcome
40	Huette P et al. Effect of a virtual reality program on patient comfort in the surgical intensive care unit of a French university hospital: study protocol for a monocentric, prospective, superiority, randomised controlled trial-ZION study. BMJ open. 2025;15(7):e101243. doi:10.1136/bmjopen-2025-101243.	Wrong outcome
41	Kao PY et al. Investigating press tack needle treatment for delirium in critically ill patients: A double-blind randomized sham-controlled pilot trial. JOURNAL OF PSYCHIATRIC RESEARCH. 2025;189:9-17. doi:10.1016/j.jpsychires.2025.05.071.	Wrong outcome
42	Mickle AM et al. Detection of postoperative delirium by family and caregivers: Evaluation of the family confusion assessment method (FAM-CAM). Journal of clinical anesthesia. 2025;106:111963. doi:10.1016/j.jclinane.2025.111963.	Wrong outcome
43	Ong B et al. Effectiveness of a noise reduction intervention in the intensive care unit: A prospective bicenter study. World journal of critical care medicine. 2025;14(4):106359. doi:10.5492/wjccm.v14.i4.106359.	Wrong outcome
44	Sadahiro R et al. Metabolome Profiling of Yokukansan in Preventing Postoperative Delirium in Elderly Cancer Patients: A Reverse Translational Study. Psychiatry and clinical neurosciences. 2025;79(10):685-696. doi:10.1111/pcn.13875.	Wrong outcome
45	Zhang H et al. Significance Analysis of the Improvement in Postoperative Recovery of Cardiac Surgery Patients Based on the Graded Management Established Using the Delirium Prediction Model. Alternative therapies in health and medicine. 2025;31(1):338-342.	Wrong outcome
46	Impact of nursing interventions on discharge disposition in patients with postoperative delirium. doi:10.1097/01.NURSE.0000978892.66327.23.	Wrong outcome
47	Fazlollah A et al. The effect of foot reflexology massage on delirium and sleep quality following cardiac surgery: A randomized clinical trial. COMPLEMENTARY THERAPIES IN MEDICINE. 2021;60. doi:10.1016/j.ctim.2021.102738.	Wrong intervention
48	Fuest KE et al. Perioperative Hemodynamic Optimization in Patients at Risk for Delirium - A Randomized-Controlled Trial. Frontiers in medicine. 2022;9:893459. doi:10.3389/fmed.2022.893459.	Wrong intervention
49	Nicklas JY et al. Personalised blood pressure management during major noncardiac surgery and postoperative neurocognitive disorders: a randomised trial. BJA open. 2024;11:100294. doi:10.1016/j.bjao.2024.100294.	Wrong intervention

50	Culshaw JR et al. Impact of Music Intervention or Usual Care on Sedative Exposure During a Spontaneous Awakening Trial among Intensive Care Unit Patients Receiving Mechanical Ventilation: A Prospective Randomized Feasibility Study. JOURNAL OF INTENSIVE CARE MEDICINE. 2025;40(11):1177-1185. doi:10.1177/08850666251343799.	Wrong intervention
51	Habicher M et al. AI-supported non-invasive measurement for intraoperative hypotension reduction in major Orthopedic and trauma surgery-study protocol for a randomized clinical trial. Trials. 2025;26(1):455. doi:10.1186/s13063-025-09194-x.	Wrong intervention
52	Kim S et al. Effects of Virtual Reality Meditation on Sleep and Delirium in ICU Patients: A Randomized Controlled Trial. Computers, informatics, nursing : CIN. 2025;43(8). doi:10.1097/CIN.0000000000001307.	Wrong intervention
53	Liu Y et al. Effects of lavender essential oil inhalation aromatherapy on postoperative sleep quality in patients with intracranial tumors: a randomized controlled trial. Frontiers in pharmacology. 2025;16:1584998. doi:10.3389/fphar.2025.1584998.	Wrong intervention
54	Nabizade SM et al. Comparing the effects of jigsaw and lecture-based education on nurses' attitudes and screening competency toward delirium in the intensive care units: A randomized controlled trial. Journal of education and health promotion. 2025;14:483. doi:10.4103/jehp.jehp_1340_24.	Wrong intervention
55	Posch MJ et al. Impact of preoperative audiovisual distraction on emergence agitation in patients undergoing orthognathic surgery: a prospective, randomized, single-blinded pilot study. Journal of dental anesthesia and pain medicine. 2025;25(6):399-408. doi:10.17245/jdapm.2025.25.6.399.	Wrong intervention
56	Sarıtaş PU et al. Evaluating oxygen reserve index-guided oxygenation for the prevention of postoperative delirium in elderly patients: a randomized controlled trial. Croatian medical journal. 2025;66(1):47-55. doi:10.3325/cmj.2025.66.47.	Wrong intervention
57	Vidal P et al. Stepped wedge cluster randomised controlled trial to assess the impact of a decision support tool for physical restraint use in intensive care units (ARBORea Study): a study protocol. BMJ open. 2025;15(5):e085674. doi:10.1136/bmjopen-2024-085674.	Wrong intervention
58	Gayatri A et al. The Effects of Nonpharmacological Interventions on Sleep Quality in Cardiac Surgical Patients: A Prospective Randomised Controlled Study. Journal of Cardiothoracic and Vascular Anesthesia. 2026;40(1):229-234. doi:10.1053/j.jvca.2025.08.036.	Wrong intervention
59	Hering TE et al. Effects of Intraoperative Prone Versus Supine Positioning on Postoperative Delirium. Geriatrics (Basel, Switzerland). 2026;11(2). doi:10.3390/geriatrics11020048.	Wrong intervention
60	Sangi S et al. Effect of Lemon Inhalation Aromatherapy on Postoperative Pain, Nausea and Vomiting, Delirium, and Inflammatory Markers After Coronary Artery Bypass Grafting: A Triple-Blind Randomized Trial. Iranian journal of pharmaceutical research : IJPR. 2026;25(1):e168770. doi:10.5812/ijpr-168770.	Wrong intervention
61	Music therapy during weaning from mechanical ventilation in the intensive care unit: Study protocol for a randomised control trial. doi:10.1080/08098131.2024.2371393.	Wrong intervention

62	Rapolthy-Beck A et al. A comparison of standard occupational therapy versus early enhanced occupation-based therapy in a medical/surgical intensive care unit: study protocol for a single site feasibility trial (EFFORT-ICU). Pilot and feasibility studies. 2021;7(1):51. doi:10.1186/s40814-021-00795-2.	Wrong population
63	Cuzco C et al. Impact of a Nurse-Driven Patient Empowerment Intervention on the Reduction in Patients' Anxiety and Depression During ICU Discharge: A Randomized Clinical Trial. Critical care medicine. 2022;50(12):1757-1767. doi:10.1097/CCM.0000000000005676.	Wrong population
64	Liao J et al. Effect of Programmed Comprehensive Nursing for Postoperative Delirium in Intensive Care Unit Patients. Evidence-based complementary and alternative medicine : eCAM. 2022;2022:1227659. doi:10.1155/2022/1227659.	Wrong population
65	Abdullah SO et al. Effect of psychoeducational program and relaxation training on critical care nurses' stress regarding care of patients with delirium. Journal of the Intensive Care Society. 2024;25(4):399-406. doi:10.1177/17511437241275307.	Wrong population
66	Lin L et al. A family intervention to prevent postoperative delirium in patients undergoing cardiac valve surgery: A randomized controlled study. Heart & Lung. 2024;63:1-8. doi:10.1016/j.hrtlng.2023.09.002.	Wrong population
67	Meidert AS et al. Effect of ice-lollies on the recovery time after anaesthesia: protocol for a cluster-randomised trial (Icesthesia). Perioperative medicine (London, England). 2026;15(1). doi:10.1186/s13741-026-00668-3.	Wrong population
68	Analysis of Preferred Music of Mechanically Ventilated Intensive Care Unit Patients Enrolled in a Randomized Controlled Trial. doi:10.1089/jicm.2021.0446.	Wrong population
69	Fu VX et al. Effect of music on clinical outcome after hip fracture operations (MCHOPIN): study protocol of a multicentre randomised controlled trial. BMJ open. 2021;11(12):e049706. doi:10.1136/bmjopen-2021-049706.	Wrong setting
70	Patiño JR et al. Efectividad de la aplicación de medidas no farmacológicas para la prevención de delirium en pacientes no ventilados ingresados en una Unidad de Cuidados Intensivos nivel ii. Acta Colombiana de Cuidado Intensivo. 2021;21(2):127-134. doi:10.1016/j.acci.2020.08.002.	Wrong setting
71	Imai K. Effect of a communication robot in the prevention of postoperative delirium in older persons: A randomized controlled trial. PLOS ONE. 2025;20(7). doi:10.1371/journal.pone.0327868.	Wrong setting
72	Kilic G et al. Effect of using eye masks and earplugs in preventing delirium in intensive care patients: A single-blinded randomized controlled trial. Journal of Critical Care. 2024;81:154570. doi:10.1016/j.jcrc.2024.154570.	Duplicate article
73	Yuyen T et al. Effectiveness of non-pharmacological intervention protocol for prevention of postoperative delirium in the surgical intensive care unit. Scientific reports. 2025;15(1):2494. doi:10.1038/s41598-025-86768-4.	Duplicate article
74	Alvarez EA et al. Non-pharmacological prevention of postoperative delirium by occupational therapy teams: A randomized clinical trial. Frontiers in medicine. 2023;10:1099594. doi:10.3389/fmed.2023.1099594.	Wrong delirium assessment method
75	Rosa RG et al. Study protocol to assess the effectiveness and safety of a flexible family visitation model for delirium prevention in adult intensive care units: a	Protocol study not meeting eligibility criteria

	cluster-randomised, crossover trial (The ICU Visits Study). BMJ OPEN. 2018;8(4). doi:10.1136/bmjopen-2017-021193.	
76	Owen VS, Sinnadurai S, Morrissey J, Colaco H, Wickson P, Dyjur D, et al. Multicentre implementation of a quality improvement initiative to reduce delirium in adult intensive care units: an interrupted time series analysis. J Crit Care. 2024; 81:154524. doi: 10.1016/j.jcrc.2024.154524.	Wrong study design
77	Fraser D, Spiva L, Forman W, Hallen C. Implementation of an early mobility program in an ICU. Am J Nurs. 2015;115(12):49-58. doi: 10.1097/01.NAJ.0000475292. 27985.fc.	Wrong study design
78	Wassenaar A et al. The impact of nursing Delirium Preventive Interventions in the Intensive Care Unit (UNDERPIN-ICU): a study protocol for a multi-centre, stepped wedge randomized controlled trial. INT J NURS STUD. 2017; 68:1–8. doi: 10.1016/j.ijnurstu.2016.11.018.	Protocol study not meeting eligibility criteria
79	Yang Q et al. Impact of an enhanced recovery after surgery program integrating cardiopulmonary rehabilitation on post-operative prognosis of patients treated with CABG: protocol of the ERAS-Care randomized controlled trial. BMC PULM MED. 2024;24(1):512. doi:10.1186/s12890-024-03286-1.	Protocol study not meeting eligibility criteria
80	Verhoeven JG et al. Study protocol for a multicenter randomized controlled trial using music prehabilitation to reduce preoperative anxiety before oncological colorectal surgery: the MU-PRIOR trial. BMJ OPEN. 2025;15(4):e095239. doi:10.1136/bmjopen-2024-095239.	Protocol study not meeting eligibility criteria
81	Yang C et al. Effect of noise isolation on postoperative delirium in elderly patients undergoing hip surgery: protocol for a randomized controlled trial. BMJ OPEN. 2025;15(4): e088092. doi:10.1136/bmjopen-2024-088092.	Protocol study not meeting eligibility criteria
82	Fiest KM et al. A study protocol for a randomized controlled trial of family-partnered delirium prevention, detection, and management in critically ill adults: the ACTIVATE study. BMC HEALTH SERV RES. 2020; 20:453. doi:10.1186/s12913-020-05281-8.	Protocol study not meeting eligibility criteria

Summary: Wrong study design (n = 19); wrong outcome (n = 24); wrong intervention (n = 15); wrong population (n = 11); wrong setting (n = 4); duplicate article (n = 2); wrong delirium assessment method (n = 1); and protocol reports not meeting eligibility criteria (n = 6). A total of 82 reports were excluded after full-text assessment. Reports with more than one exclusion reason in Rayyan were assigned one mutually exclusive primary reason. For 22 reports without an exclusion reason exported from Rayyan, the primary reason was assigned after reassessment of the available full-text publication against the predefined eligibility criteria.

Table S5. Sensitivity analyses of postoperative delirium incidence

Panel A. Leave-one-out sensitivity analysis using a random-effects model

Study removed	Pooled RR	95% CI lower	95% CI upper	τ^2 (REML)	I^2 (%)	Heterogeneity p
None — primary analysis	0.611	0.481	0.776	0.04934	25.2	0.182
Topal 2025	0.615	0.484	0.782	0.04886	29.1	0.152
Esfahanian 2022	0.633	0.500	0.801	0.04092	20.7	0.234
Kappen 2023	0.611	0.477	0.783	0.05485	30.8	0.137
Abbasinia 2021	0.613	0.480	0.782	0.05182	30.7	0.138
Jiang 2024	0.596	0.449	0.792	0.07741	31.0	0.136
Méndez-Martínez 2024	0.632	0.501	0.797	0.03988	8.1	0.365
Kim 2020	0.607	0.476	0.775	0.05298	30.8	0.137
Xue 2020	0.626	0.489	0.802	0.04959	27.2	0.170
Zhang 2017	0.648	0.507	0.827	0.03658	20.9	0.232
Faustino 2022	0.611	0.472	0.792	0.06101	30.6	0.139
Moon 2015	0.606	0.464	0.791	0.06673	30.8	0.137
Giraud 2016	0.576	0.454	0.731	0.03498	12.2	0.322
Lei 2017	0.573	0.462	0.712	0.00870	4.3	0.403
Kitisin 2025	0.608	0.469	0.788	0.06180	30.9	0.137

Panel B. Sensitivity analysis restricted to randomized controlled trials

Analysis	Studies	Participants	Intervention events/total	Control events/total	Pooled RR	95% CI	I^2	τ^2
Random-effects analysis restricted to randomized controlled trials	13	1,988	128/1,014	202/974	0.65	0.51–0.83	20.9%	0.0366

Note: Zhang et al. (2017) was excluded because it used a non-randomized prospective before–after design. All other studies contributing to the revised primary incidence meta-analysis were randomized controlled trials. The analysis used the same random-effects model, REML estimator, continuity correction, and confidence interval method as the primary analysis.

Panel C. Sensitivity analyses according to methodological quality and statistical inference

Analysis	Studies	Participants	Pooled RR	95% CI	I^2	τ^2
Primary analysis	14	2,266	0.61	0.48–0.78	25.2%	0.0493
RCTs only	13	1,988	0.65	0.51–0.83	20.9%	0.0366
Low risk + some concerns only	9	1,573	0.70	0.55–0.89	7.9%	0.0190
Low risk	4	834	0.68	0.46–0.99	46.0%	0.0577
Some concerns	5	739	0.71	0.49–1.04	0%	0.0207

High risk	4	415	0.37	0.19–0.73	8.4%	≈0
Serious risk, ROBINS-I	1	278	0.45	0.28–0.74	NA	NA
REML + Hartung–Knapp	14	2,266	0.61	0.47–0.79	25.2%	0.0493
Clearly postoperative/surgical populations only	12	1,999	0.60	0.45–0.81	36.2%	0.0839

Note: Risk-of-bias categories for randomized trials were based on overall RoB 2 judgments. The single non-randomized study contributing to the revised primary meta-analysis, Zhang et al. (2017), was assessed using ROBINS-I and judged to have serious overall risk of bias. The analysis restricted to clearly postoperative or surgical populations excluded Moon et al. (2015) and Faustino et al. (2022), which enrolled broader critically ill ICU populations. The Hartung–Knapp analysis retained the REML estimate of between-study variance.

Table S6. GRADE evidence profile for postoperative delirium incidence

Outcome	Studies/design	Participants	Relative effect	Absolute effect	Risk of bias	Inconsistency	Indirectness	Imprecision	Publication bias	Overall certainty
Postoperative delirium incidence	14 studies: 13 RCTs and 1 non-randomized study	2,266	RR 0.61 (95% CI 0.48–0.78)	85 fewer per 1000 (49–113 fewer)	Serious	Not serious	Not serious	Not serious	Not serious	⊕⊕⊕◯ Moderate

Note:

a. Risk of bias: Downgraded one level because four randomized studies contributing to the primary meta-analysis were judged to have high overall risk of bias, and the single non-randomized study was judged to have serious overall risk of bias using ROBINS-I.

b. Inconsistency: Not downgraded because heterogeneity was low to moderate ($I^2 = 25.2\%$; $\tau^2 = 0.0493$), and the overall direction of effect was consistent.

c. Indirectness: Not downgraded because most contributing populations were postoperative or surgical ICU populations, and a sensitivity analysis restricted to clearly postoperative/surgical studies produced a consistent effect (RR = 0.60; 95% CI: 0.45–0.81).

d. Imprecision: Not downgraded because the 95% confidence interval excluded the null effect and the sample size was substantial.

e. Publication bias: Egger's test suggested possible small-study effects (p = 0.068), but the exploratory trim-and-fill analysis remained statistically significant (adjusted RR = 0.73; 95% CI: 0.57–0.93).

f. Sensitivity: Restriction to 13 randomized controlled trials produced RR = 0.65 (95% CI: 0.51–0.83).

Table S7. Comparison between prospectively registered PROSPERO Version 1.0 (19 February 2026) and the final review methods and protocol amendments

Domain	PROSPERO registration	Final review	Classification / explanation
Population age	Overarching PICOS: adults ≥18 y; separate eligibility field: 18–59 y	Adults ≥18 y, no upper age restriction	Clarification of internally inconsistent registration fields
Study design	PICOS: RCTs, quasi-experimental and comparative cohort studies; separate field: randomized studies only	RCTs plus eligible prospective comparative non-randomized, quasi-experimental and before–after studies	Clarification of internally inconsistent registration fields
Search dates	Eligibility field: 2020–2025; search section: no date restriction	1 Jan 2014–29 May 2026	Protocol deviation/clarification; lower limit selected to capture contemporary post-2013 ICU delirium-prevention framework
Delirium instruments	CAM-ICU/ICDSC; outcome section also allowed equivalent validated assessments	Validated delirium instruments or diagnostic criteria, including CAM-ICU, CAM, DOSS/DSM-5, Nu-DESC, DDS, 4AT and ICDSC	Clarification consistent with PROSPERO wording permitting equivalent validated tools
Risk-of-bias tools	RoB 1 and RoB 2	RoB 2 for RCTs; ROBINS-I for non-randomized studies	Methodological update according to study design
Primary synthesis	Random-effects meta-analysis; RR for dichotomous outcomes	Random-effects REML meta-analysis using RR	Prespecified
Heterogeneity	I^2	I^2 and τ^2	Prespecified with additional reporting of τ^2
Intervention subgroup analysis	Type of intervention	Intervention category	Prespecified
Other planned subgroup analyses	Surgery type, age, frailty, adherence	Not performed	Insufficient and inconsistent study-level reporting / sparse strata
Risk-of-bias sensitivity	Exclusion of high-risk studies	Exclusion of high RoB 2 and serious ROBINS-I studies	Prespecified; operationalized according to final bias tools
Extreme-weight sensitivity	Exclusion of studies with extreme weight proposed	Leave-one-out analysis used instead	Post hoc methodological modification
RCT-only sensitivity	Not specifically registered	Performed	Post hoc
RoB-stratified subgroup analysis	Not registered	Low/some concerns/high RoB 2	Exploratory post hoc
Hartung–Knapp	Not registered	Performed	Post hoc during peer review
Funnel/Egger and trim-and-fill	Not specifically prespecified in statistical plan	Performed	Exploratory post hoc analyses of small-study effects

Outcome-selection hierarchy	Not explicitly registered	Applied uniformly to multiple instruments/timepoints	Post hoc clarification during revision
Postoperative/surgical-only sensitivity	Not registered	Performed	Post hoc sensitivity analysis addressing population indirectness
GRADE	Planned	Performed	Prespecified
Study identification methods	Forward citation searching, backward reference checking, and trial/study register searching were planned	Study identification was based on the reported electronic information sources; these additional methods were not performed	Protocol deviation
Contact with study investigators	Authors would be contacted when required data were unavailable	Investigators were not contacted; unavailable characteristics were recorded as NR and not imputed	Protocol deviation
Data extraction process	Data extraction independently by at least two reviewers	Data extracted by one reviewer and checked by a second reviewer, with discrepancies resolved by consensus	Protocol deviation / methodological clarification
Population/setting scope	Postoperative adults admitted to ICU/critical care following major surgery	A small number of broader critically ill ICU populations were retained when the intervention and delirium-related outcome were directly relevant; influence assessed in a postoperative/surgical-only sensitivity analysis	Protocol deviation; addressed by post hoc sensitivity analysis

Note: Prespecified status was determined against PROSPERO Version 1.0. Subsequent updates published on 26 August 2026 documented the completed review methods and amendments and were not used to classify analyses as prospectively specified.
